# Supplementary figures and images for: Downregulation of Dickkopf-3, a Wnt antagonist elevated in Alzheimer’s disease, restores synapse integrity and memory in a disease mouse model
Source: eLife. 2024 Jan 29;12:RP89453. doi: 10.7554/eLife.89453 (PMC10945611; doi:10.7554/eLife.89453)

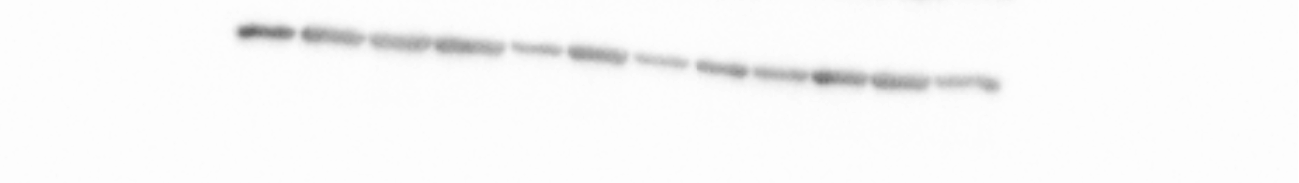

Supplement: Figure 1—source data 1. — Raw and annotated WB images. The representative western blot images for Figure 1B and C are indicated within a blue square. [file elife-89453-fig1-data1.zip › WB Figure 1/Figure 1B/Fig 1B Actin.tif]

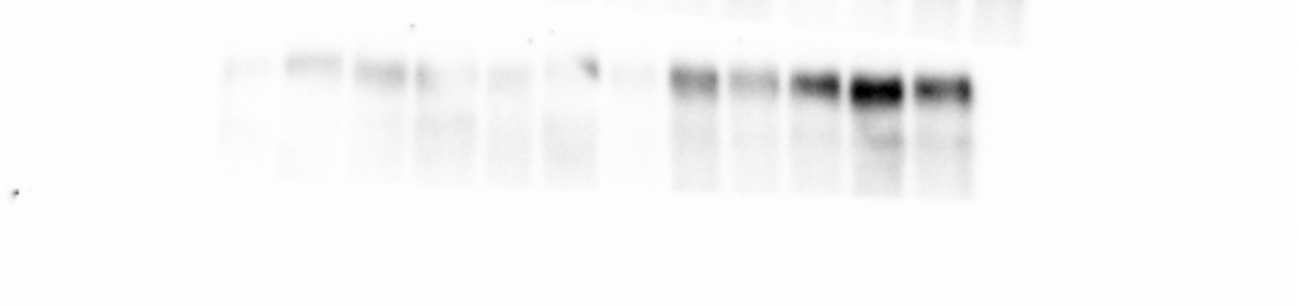

Supplement: Figure 1—source data 1. — Raw and annotated WB images. The representative western blot images for Figure 1B and C are indicated within a blue square. [file elife-89453-fig1-data1.zip › WB Figure 1/Figure 1B/Fig 1B Dkk3.tif]

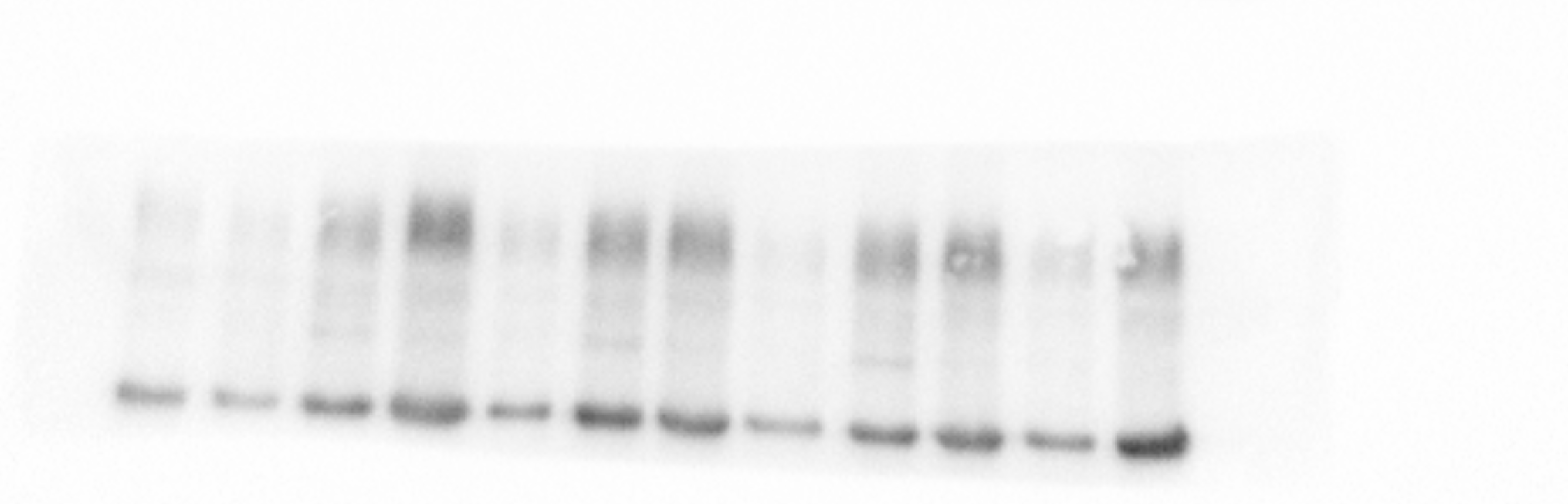

Supplement: Figure 1—source data 1. — Raw and annotated WB images. The representative western blot images for Figure 1B and C are indicated within a blue square. [file elife-89453-fig1-data1.zip › WB Figure 1/Figure 1C/Fig 1C Actin.tif]

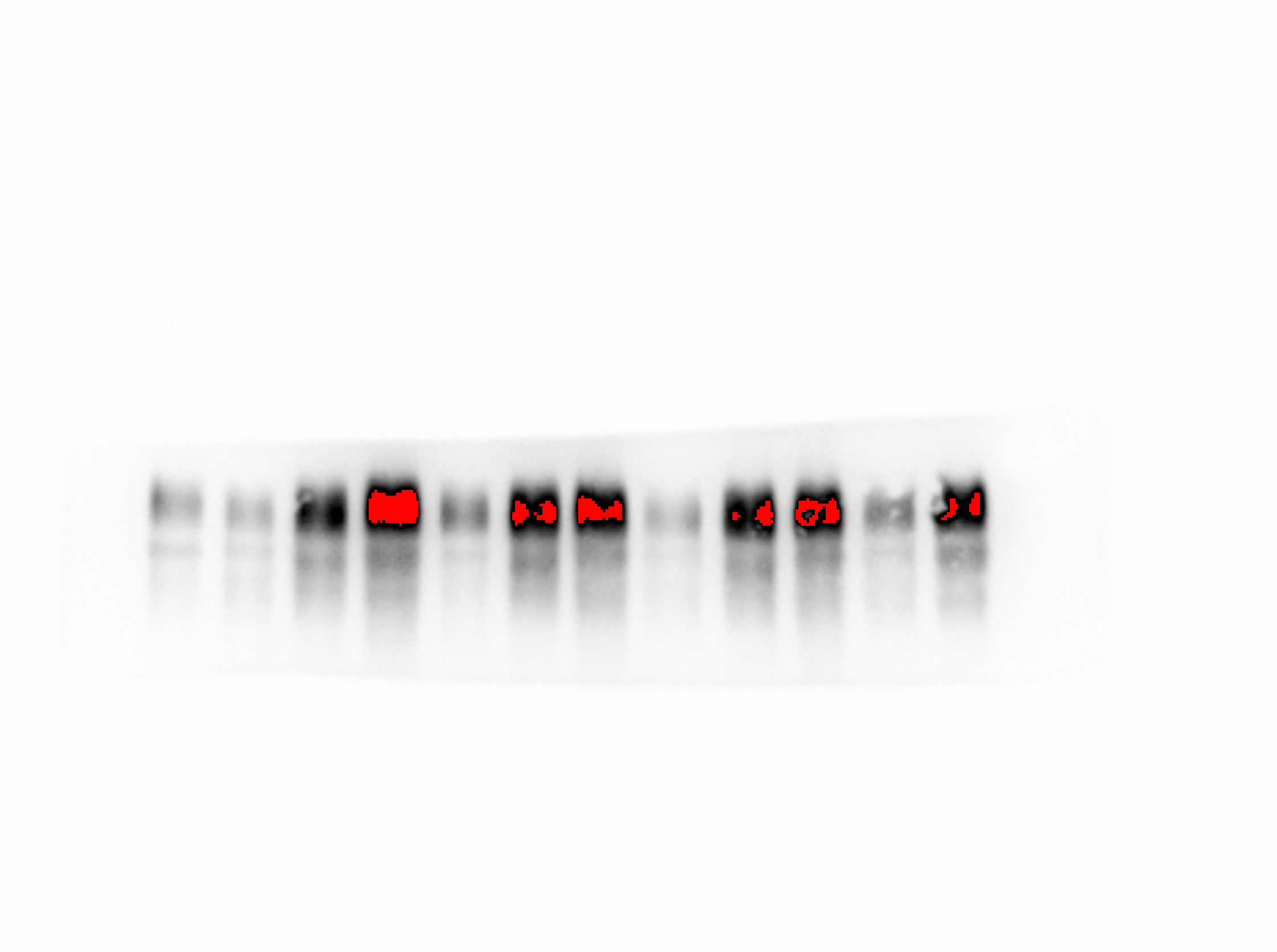

Supplement: Figure 1—source data 1. — Raw and annotated WB images. The representative western blot images for Figure 1B and C are indicated within a blue square. [file elife-89453-fig1-data1.zip › WB Figure 1/Figure 1C/Fig 1C Dkk3.tif]

**Figure 1B**

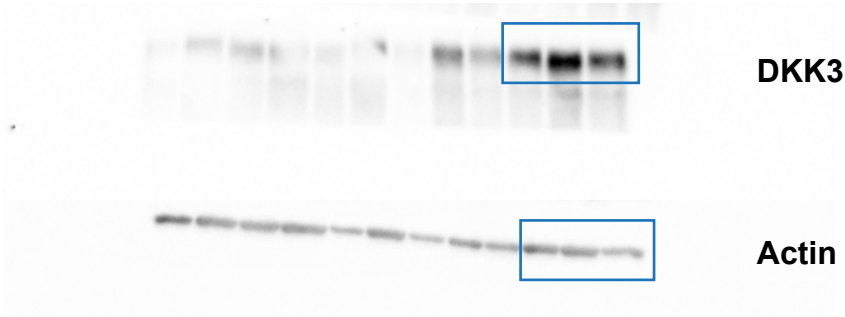

**Figure 1C**

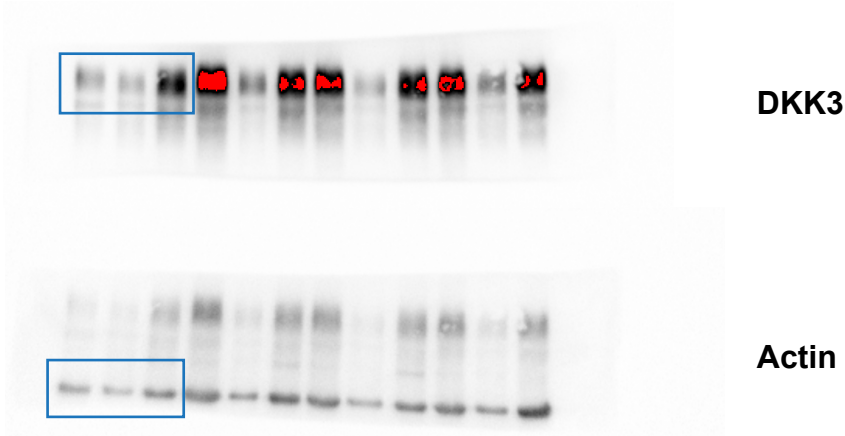

Supplement: Figure 1—source data 1. — Raw and annotated WB images. The representative western blot images for Figure 1B and C are indicated within a blue square. [file elife-89453-fig1-data1.zip › WB Figure 1/Source data_Figure 1.pdf]

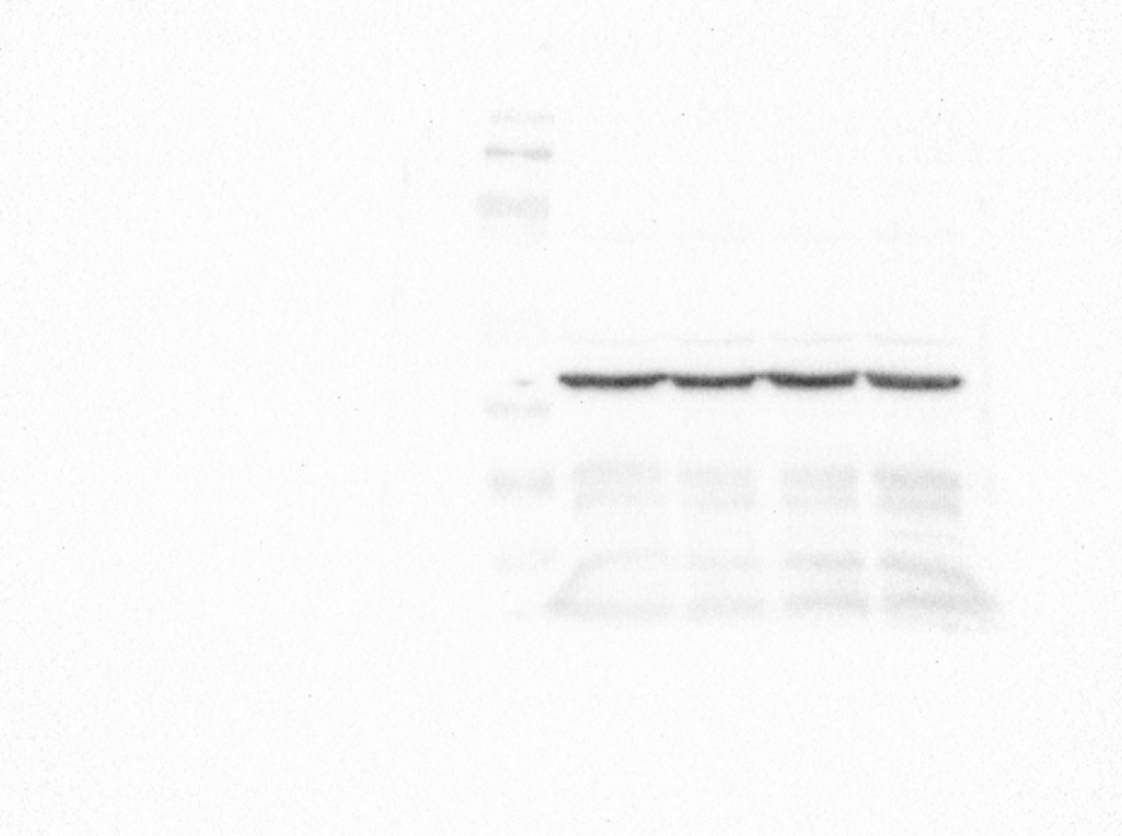

Supplement: Figure 2—source data 1. — Raw and annotated WB images. The representative western blot images for Figure 2E and F are indicated within a blue square. [file elife-89453-fig2-data1.zip › WB Figure 2/Figure 2E/Fig 2E Cell lysate actin.tif]

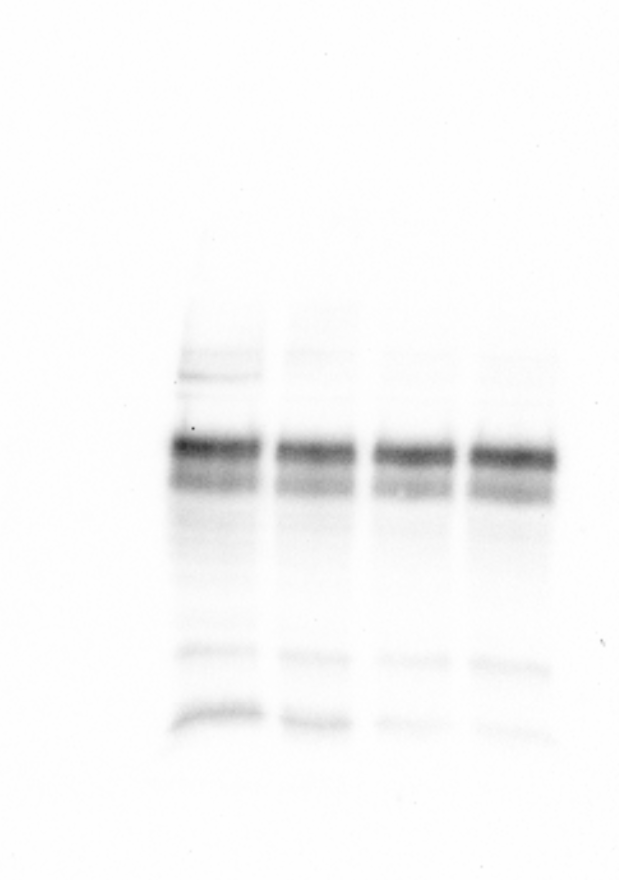

Supplement: Figure 2—source data 1. — Raw and annotated WB images. The representative western blot images for Figure 2E and F are indicated within a blue square. [file elife-89453-fig2-data1.zip › WB Figure 2/Figure 2E/Fig 2E Cell lysate Dkk3.tif]

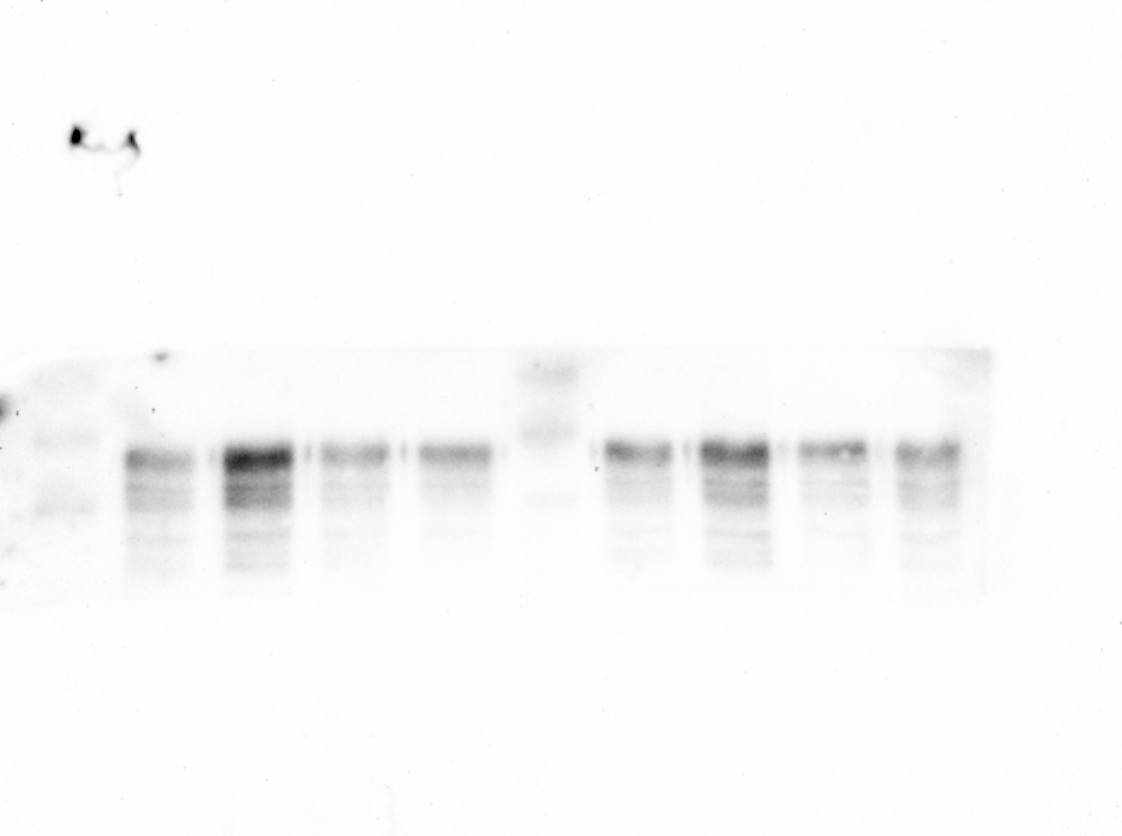

Supplement: Figure 2—source data 1. — Raw and annotated WB images. The representative western blot images for Figure 2E and F are indicated within a blue square. [file elife-89453-fig2-data1.zip › WB Figure 2/Figure 2E/Fig 2E Extracell Dkk3.tif]

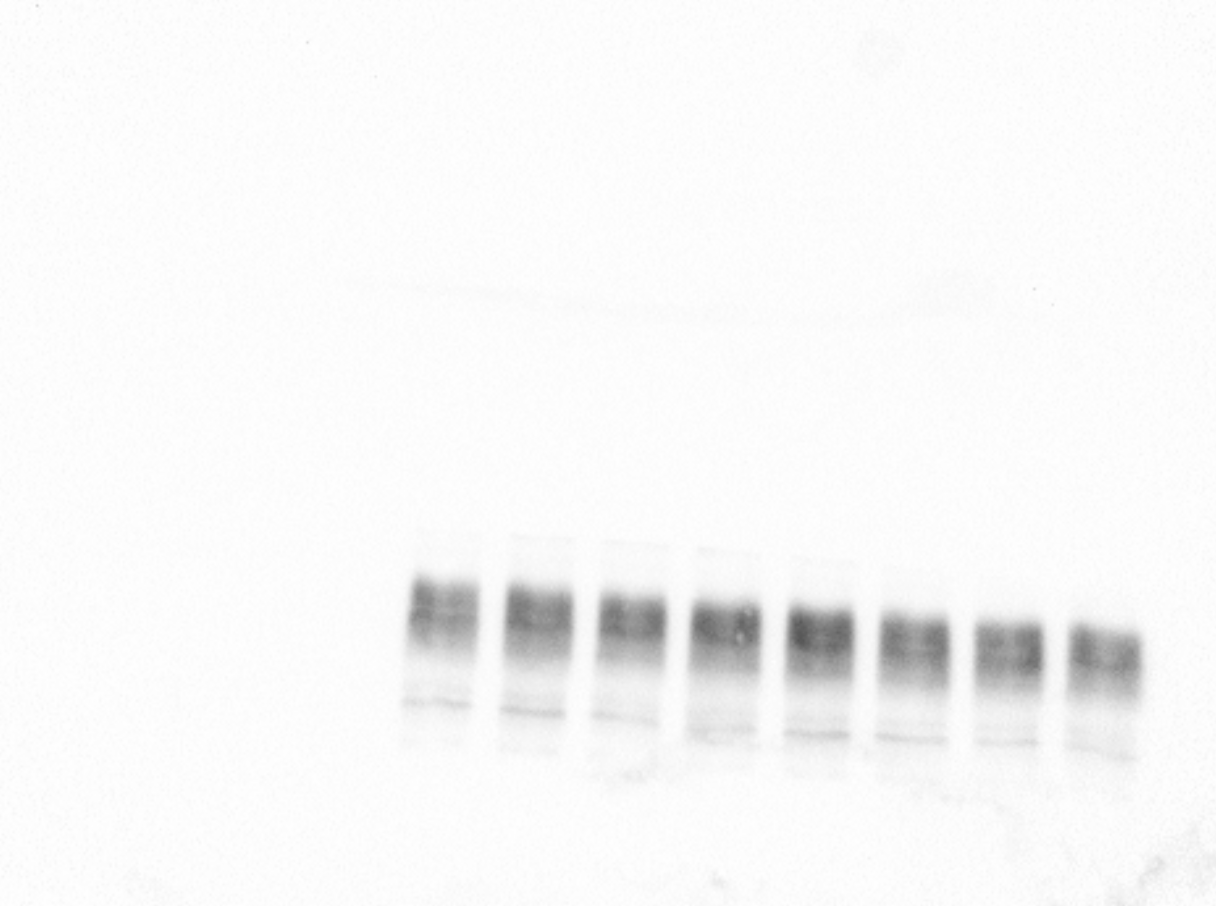

Supplement: Figure 2—source data 1. — Raw and annotated WB images. The representative western blot images for Figure 2E and F are indicated within a blue square. [file elife-89453-fig2-data1.zip › WB Figure 2/Figure 2F/Fig 2F Cell lysate Dkk3.tif]

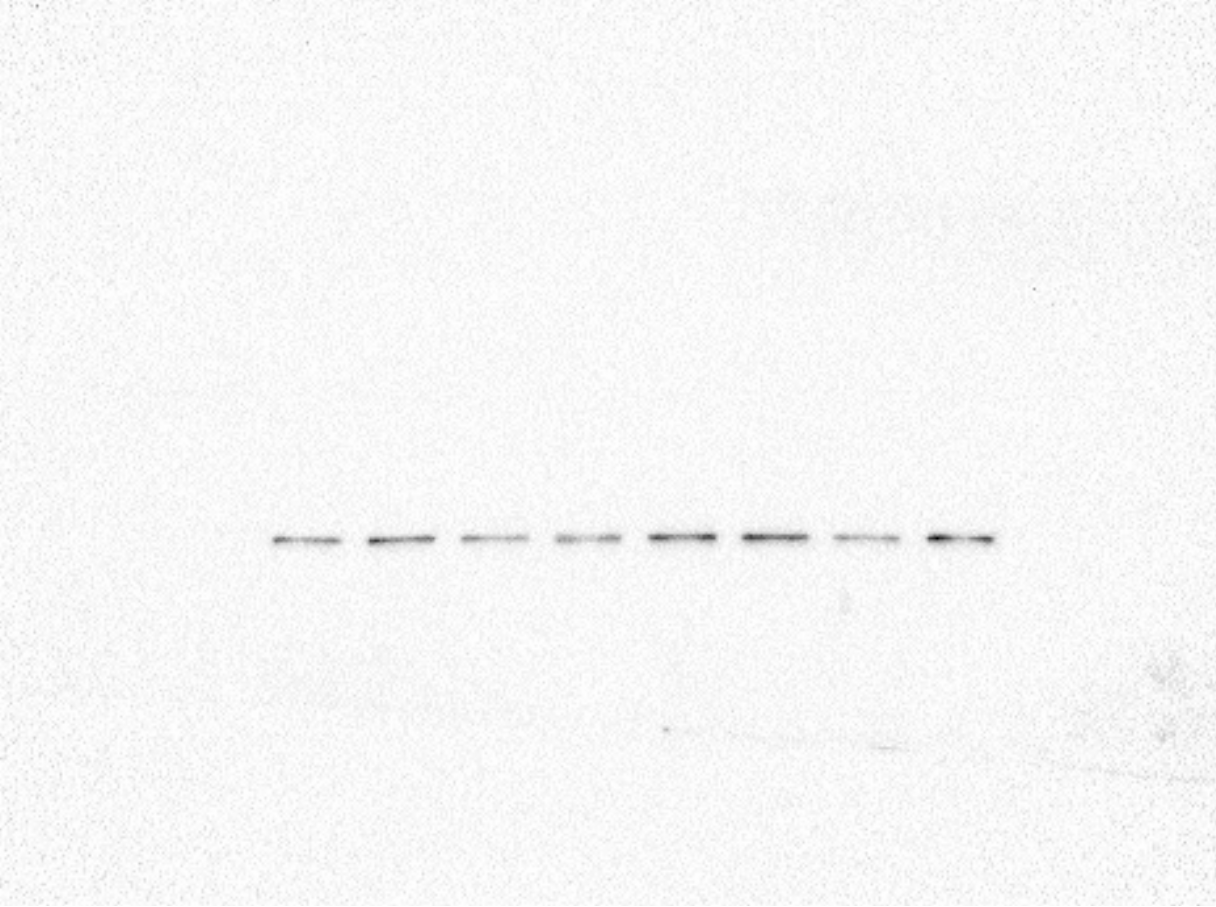

Supplement: Figure 2—source data 1. — Raw and annotated WB images. The representative western blot images for Figure 2E and F are indicated within a blue square. [file elife-89453-fig2-data1.zip › WB Figure 2/Figure 2F/Fig 2F Cell lysate Vinculin.tif]

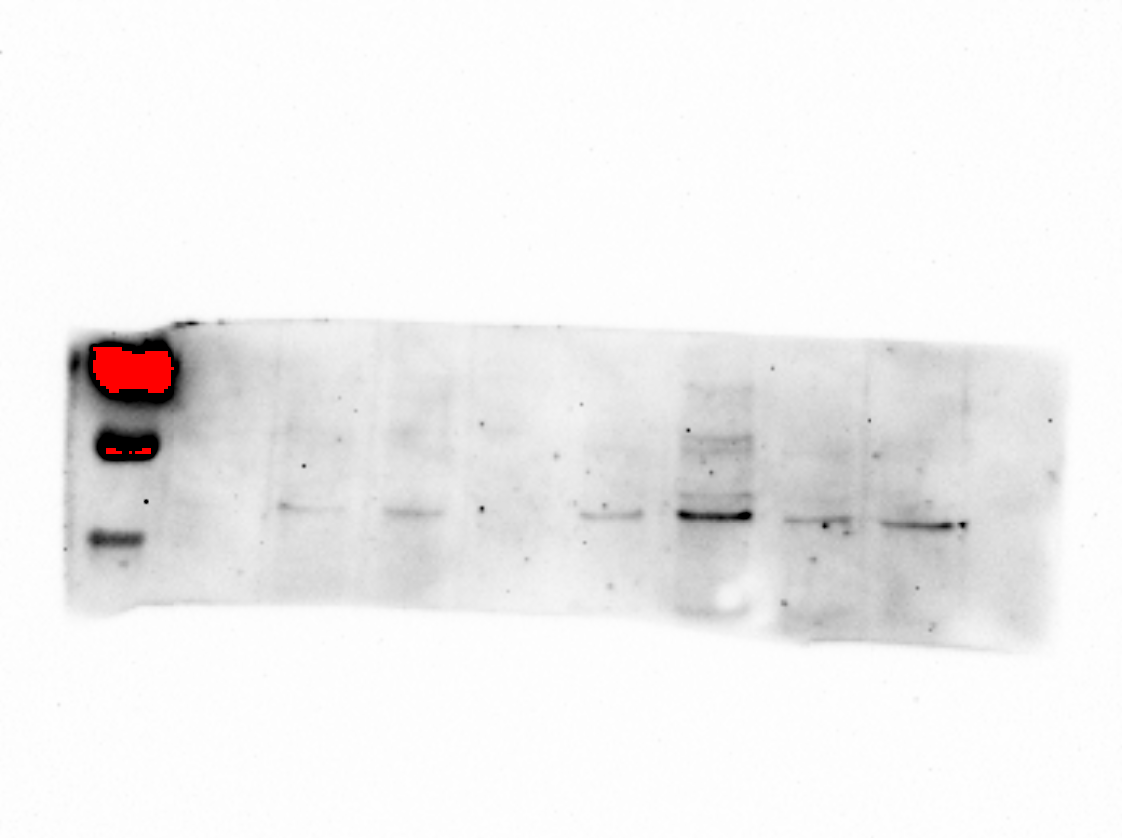

Supplement: Figure 2—source data 1. — Raw and annotated WB images. The representative western blot images for Figure 2E and F are indicated within a blue square. [file elife-89453-fig2-data1.zip › WB Figure 2/Figure 2F/Fig 2F Extracell Dkk3.tif]

**Figure 2E**

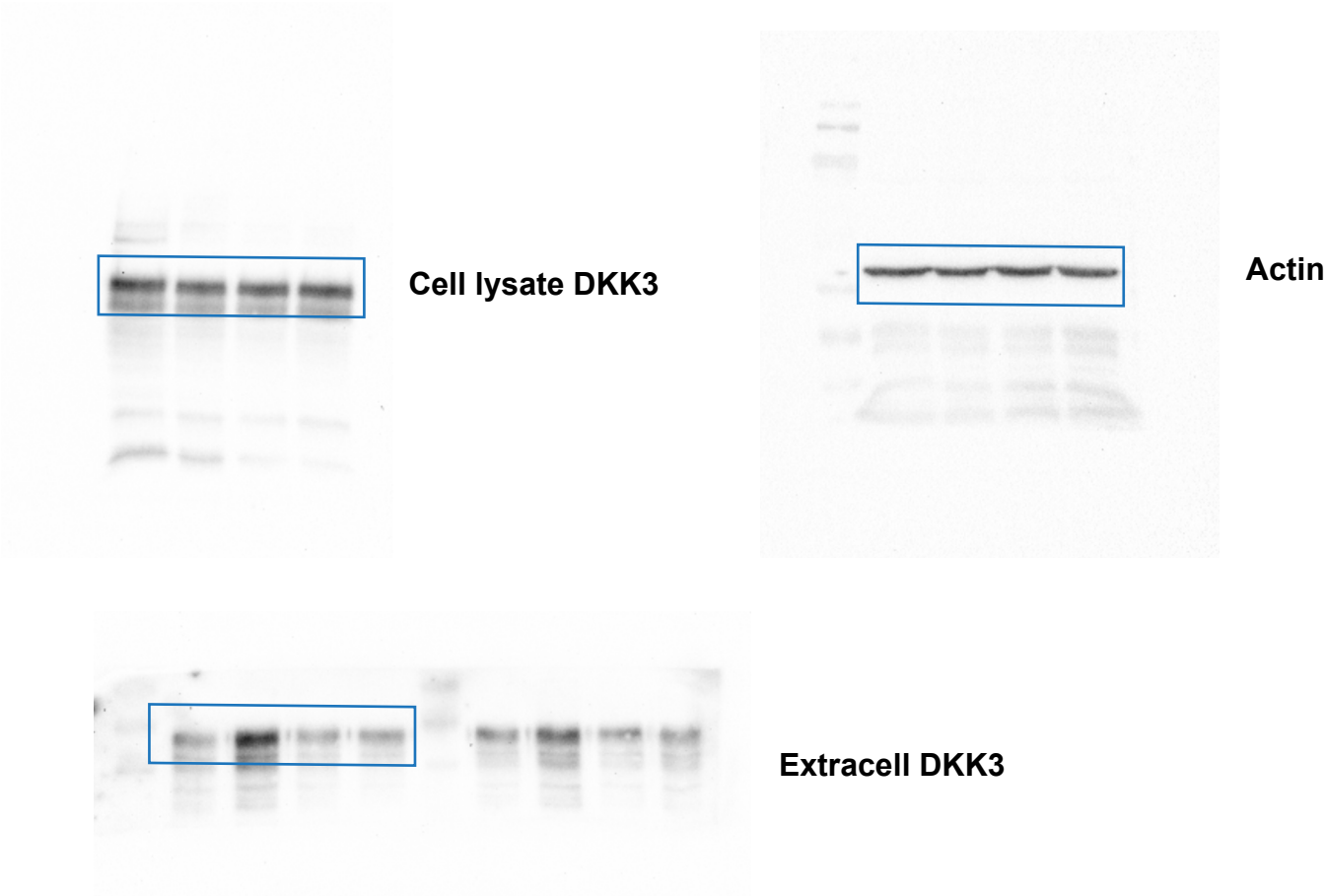

**Figure 2F**

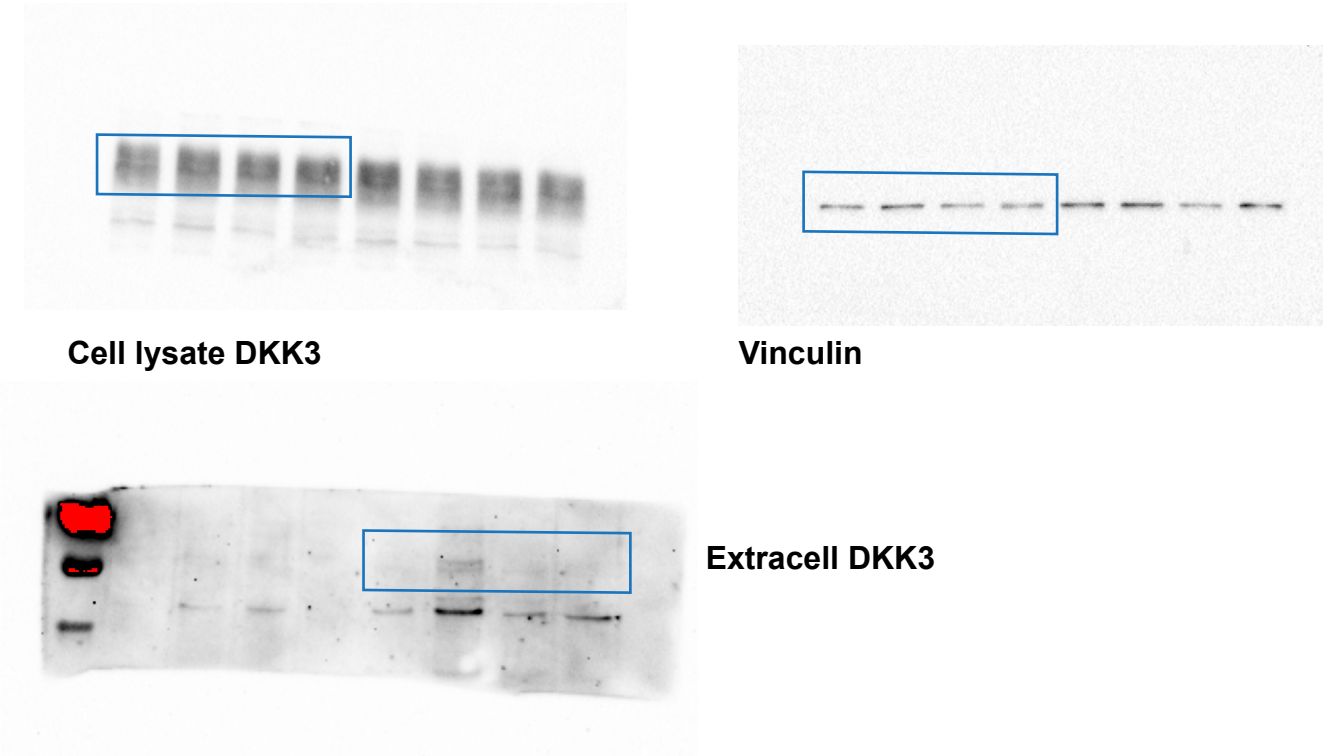

Supplement: Figure 2—source data 1. — Raw and annotated WB images. The representative western blot images for Figure 2E and F are indicated within a blue square. [file elife-89453-fig2-data1.zip › WB Figure 2/Source data_Figure 2.pdf]

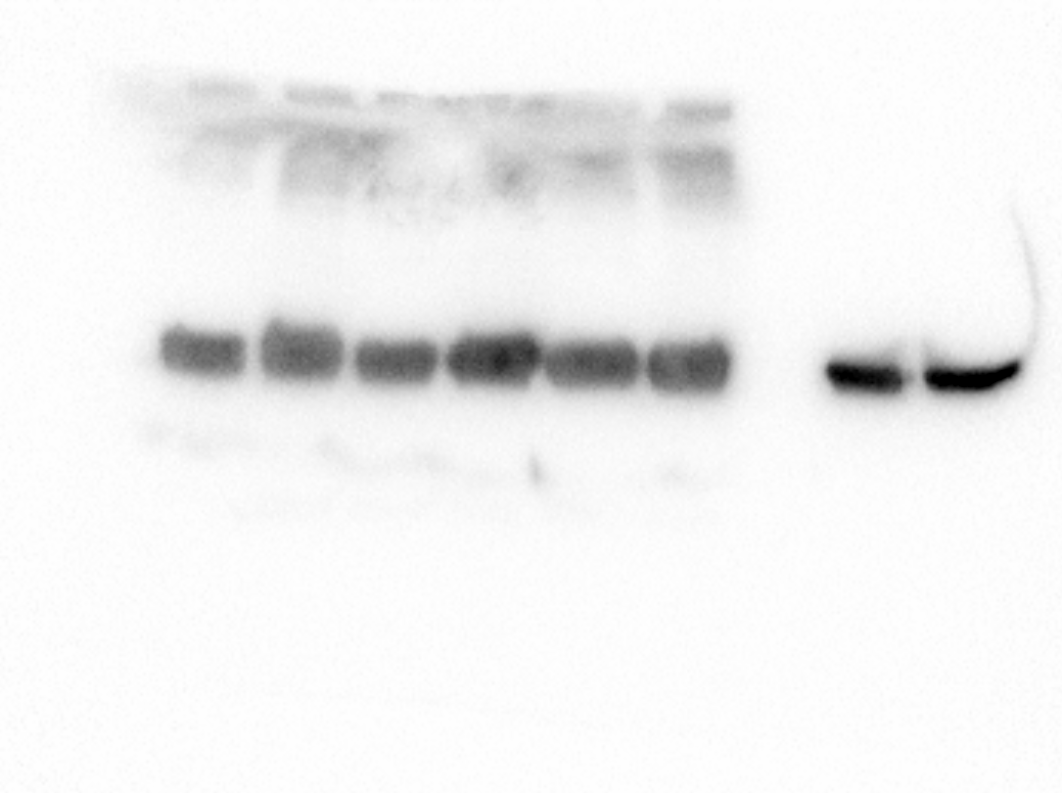

Supplement: Figure 2—figure supplement 1—source data 1. — Raw and annotated WB images. The representative western blot images for Figure 2—figure supplement 1A are indicated within a blue square. [file elife-89453-fig2-figsupp1-data1.zip › WB Figure 2 - figure supplement 1/Fig2-S1A Actin.tif]

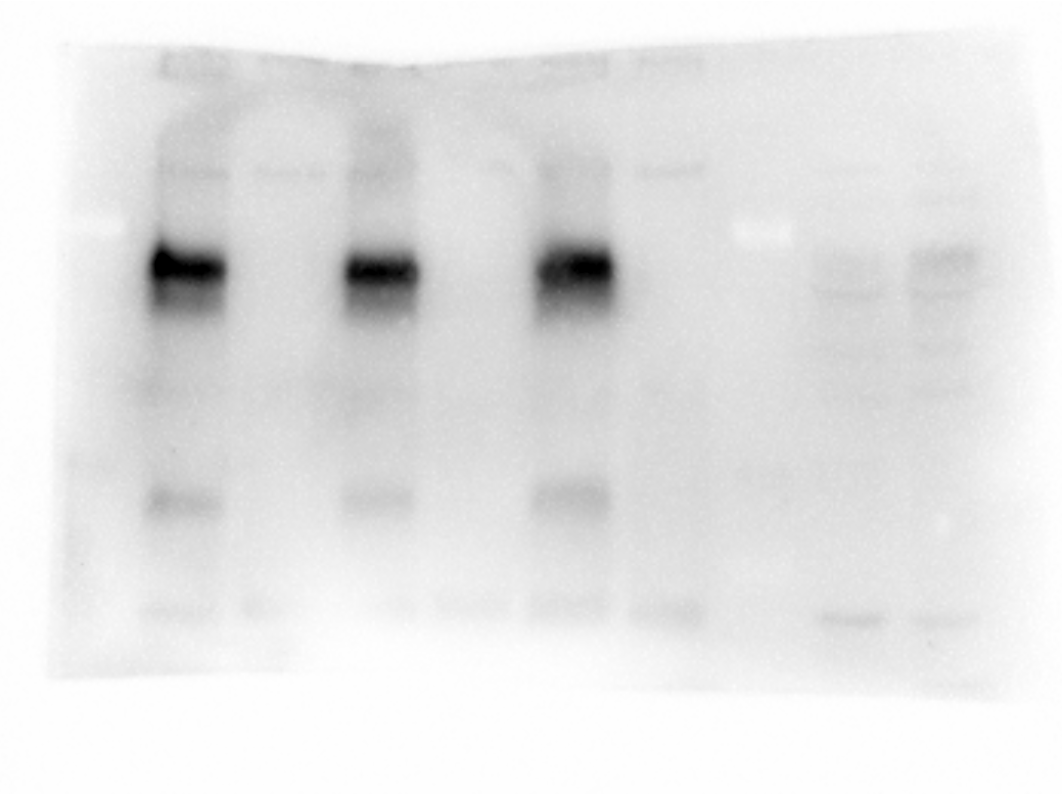

Supplement: Figure 2—figure supplement 1—source data 1. — Raw and annotated WB images. The representative western blot images for Figure 2—figure supplement 1A are indicated within a blue square. [file elife-89453-fig2-figsupp1-data1.zip › WB Figure 2 - figure supplement 1/Fig2-S1A Dkk3.tif]

**Figure 2 - figure supplement 1A**

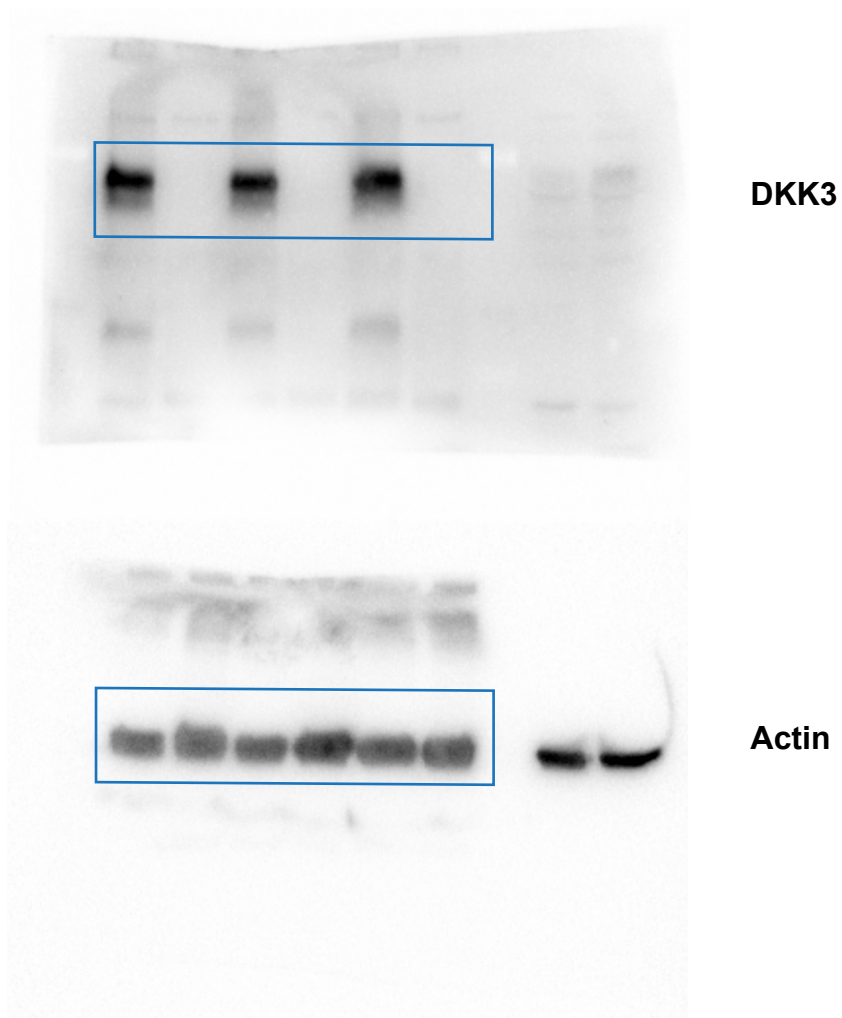

Supplement: Figure 2—figure supplement 1—source data 1. — Raw and annotated WB images. The representative western blot images for Figure 2—figure supplement 1A are indicated within a blue square. [file elife-89453-fig2-figsupp1-data1.zip › WB Figure 2 - figure supplement 1/Source data_Figure 2 - figure supplement 1.pdf]

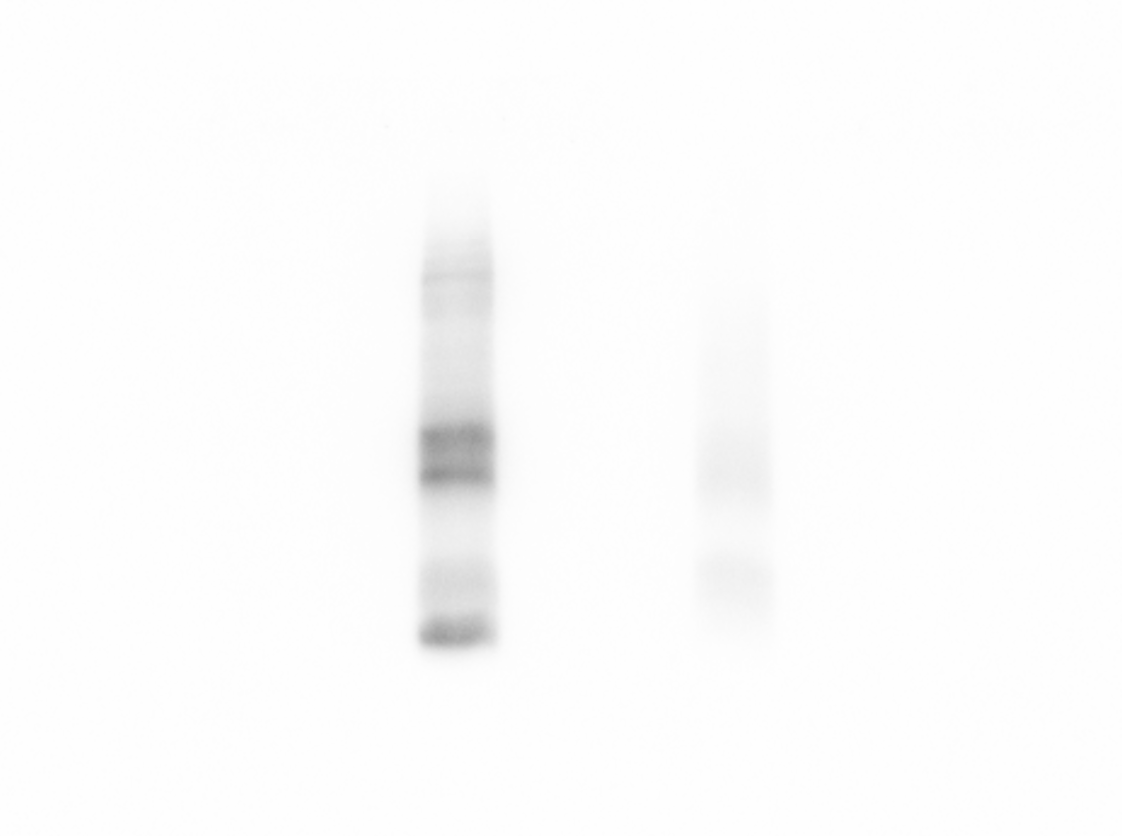

Supplement: Figure 2—figure supplement 2—source data 1. — Raw and annotated WB images. The representative western blot images for Figure 2—figure supplement 2F–G are indicated within a blue square. [file elife-89453-fig2-figsupp2-data1.zip › WB Figure 2 - figure supplement 2/Figure 2 - figure supplement 2F/Fig2-S2F.tif]

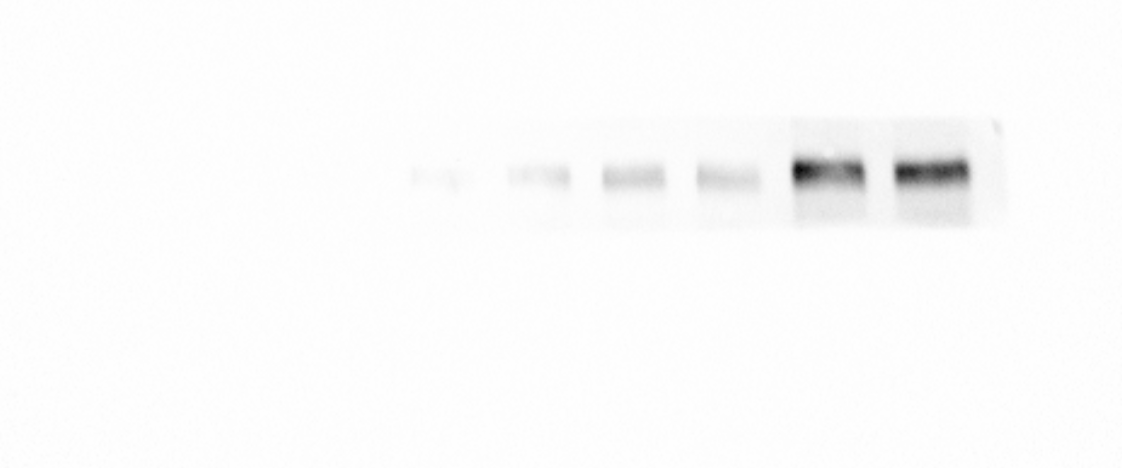

Supplement: Figure 2—figure supplement 2—source data 1. — Raw and annotated WB images. The representative western blot images for Figure 2—figure supplement 2F–G are indicated within a blue square. [file elife-89453-fig2-figsupp2-data1.zip › WB Figure 2 - figure supplement 2/Figure 2 - figure supplement 2G/Fig2-S2G Cell lysate Dkk3.tif]

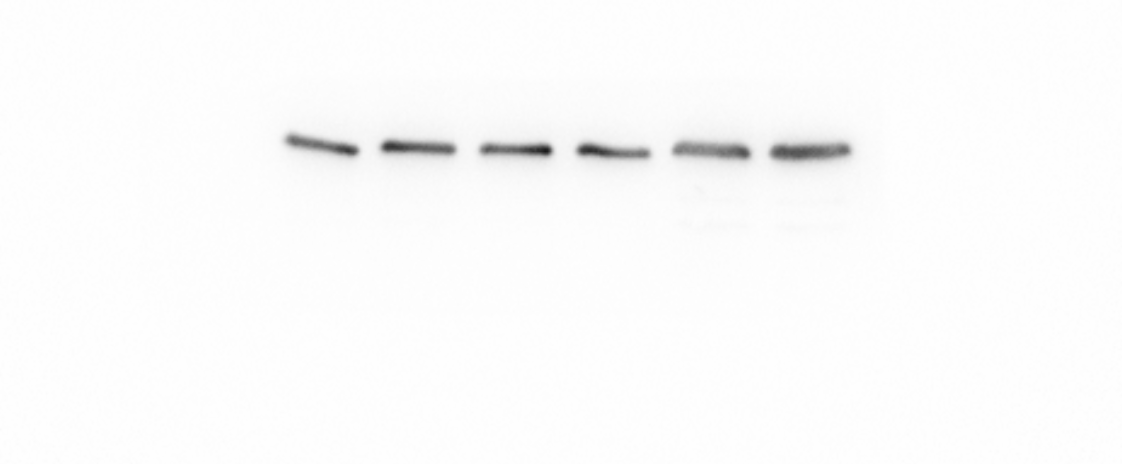

Supplement: Figure 2—figure supplement 2—source data 1. — Raw and annotated WB images. The representative western blot images for Figure 2—figure supplement 2F–G are indicated within a blue square. [file elife-89453-fig2-figsupp2-data1.zip › WB Figure 2 - figure supplement 2/Figure 2 - figure supplement 2G/Fig2-S2G Cell lysate Gapdh.tif]

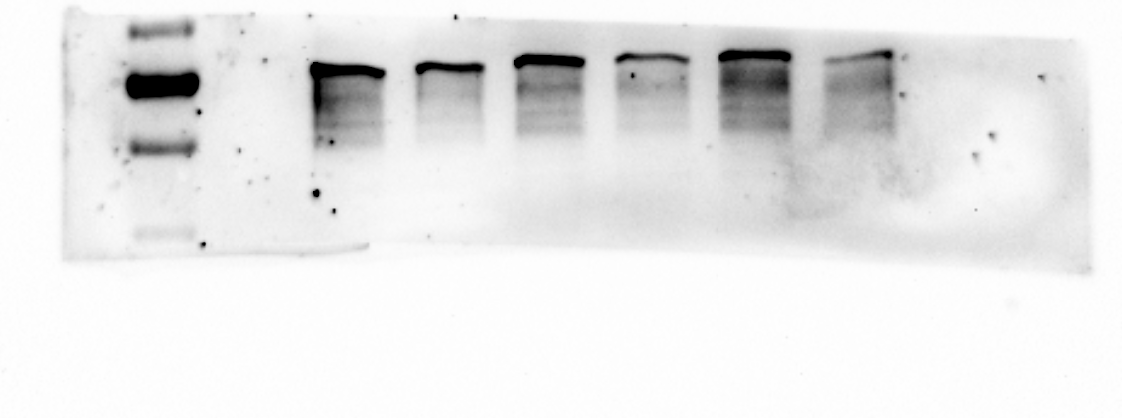

Supplement: Figure 2—figure supplement 2—source data 1. — Raw and annotated WB images. The representative western blot images for Figure 2—figure supplement 2F–G are indicated within a blue square. [file elife-89453-fig2-figsupp2-data1.zip › WB Figure 2 - figure supplement 2/Figure 2 - figure supplement 2G/Fig2-S2G Extracell Dkk3.tif]

**Figure 2 - figure supplement 2F**

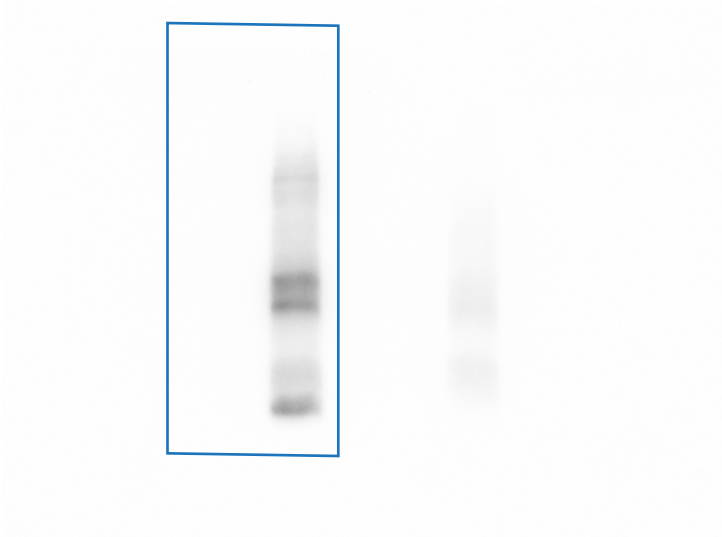

**6E10**

**Figure 2 - figure supplement 2G**

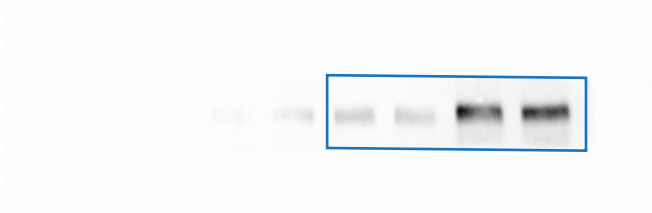

**Cell lysate DKK3**

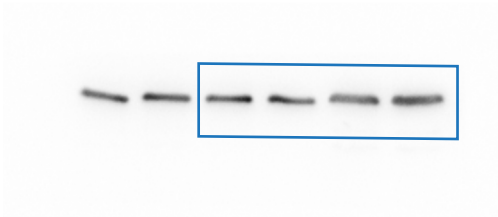

**Actin**

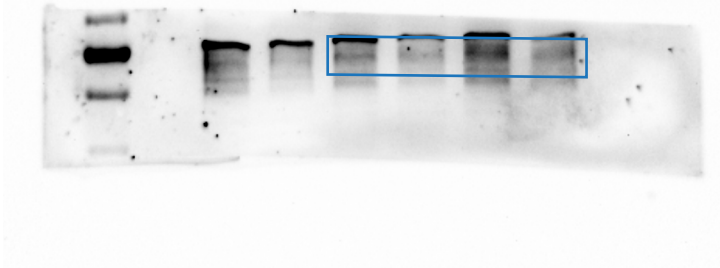

**Extracell DKK3**

Supplement: Figure 2—figure supplement 2—source data 1. — Raw and annotated WB images. The representative western blot images for Figure 2—figure supplement 2F–G are indicated within a blue square. [file elife-89453-fig2-figsupp2-data1.zip › WB Figure 2 - figure supplement 2/Source data_Figure 2 - figure supplement 2.pdf]

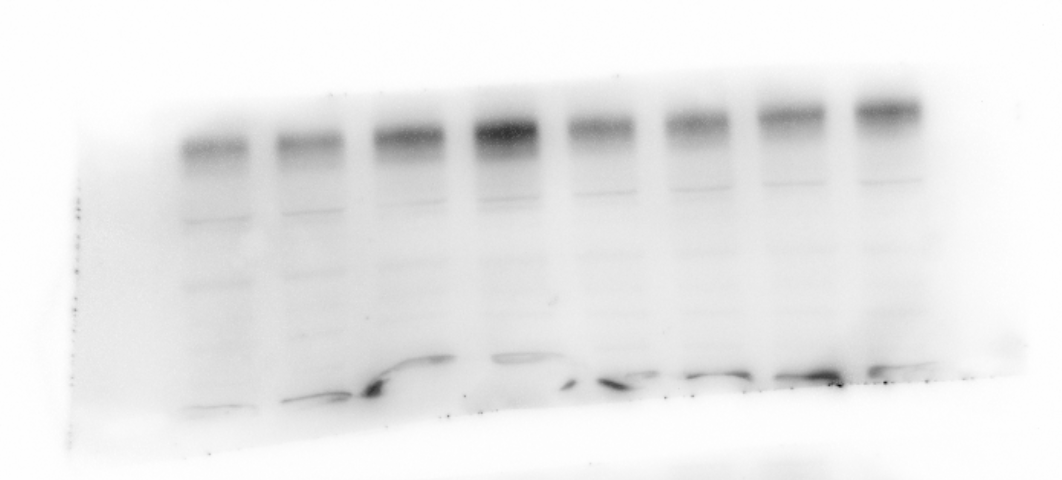

Supplement: Figure 2—figure supplement 3—source data 1. — Raw and annotated WB images. The representative western blot images for Figure 2—figure supplement 3A–D are indicated within a blue square. [file elife-89453-fig2-figsupp3-data1.zip › WB Figure 2 - figure supplement 3/Figure 2 - figure supplement 3A/Fig2-S3A Cell lysate Dkk3.tif]

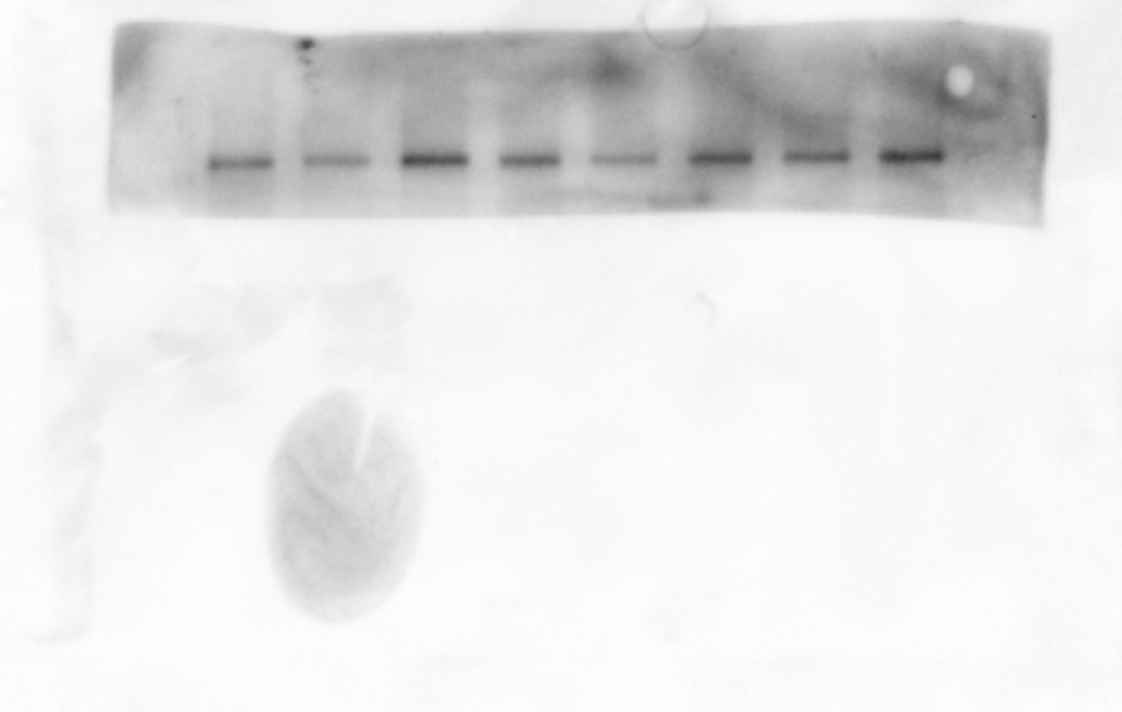

Supplement: Figure 2—figure supplement 3—source data 1. — Raw and annotated WB images. The representative western blot images for Figure 2—figure supplement 3A–D are indicated within a blue square. [file elife-89453-fig2-figsupp3-data1.zip › WB Figure 2 - figure supplement 3/Figure 2 - figure supplement 3A/Fig2-S3A Cell lysate pGluA1.tif]

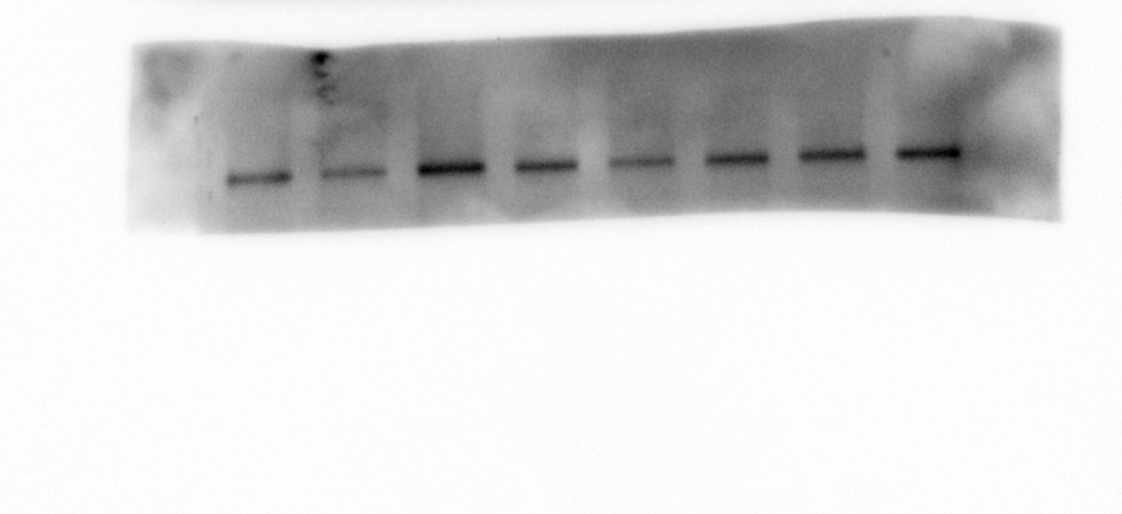

Supplement: Figure 2—figure supplement 3—source data 1. — Raw and annotated WB images. The representative western blot images for Figure 2—figure supplement 3A–D are indicated within a blue square. [file elife-89453-fig2-figsupp3-data1.zip › WB Figure 2 - figure supplement 3/Figure 2 - figure supplement 3A/Fig2-S3A Cell lysate tGluA1.tif]

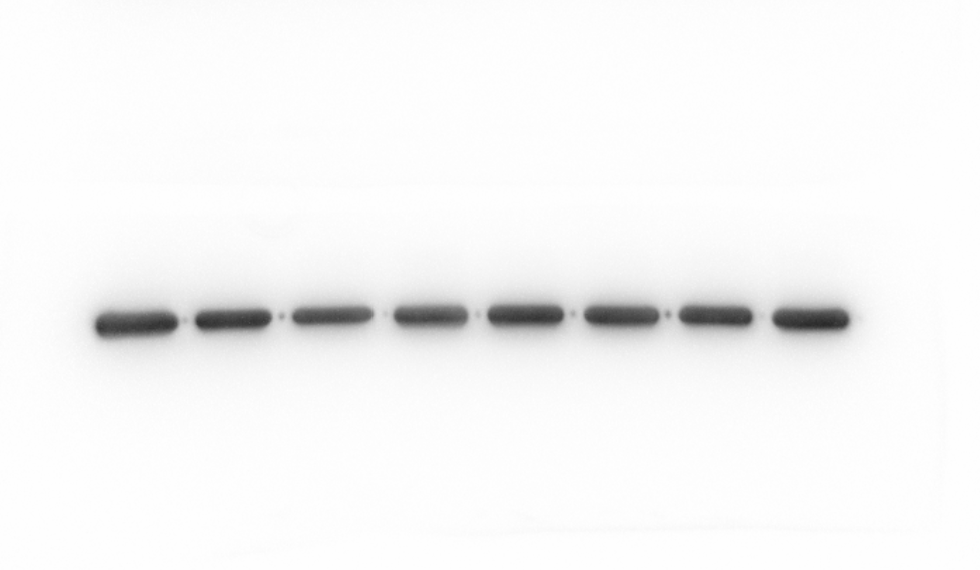

Supplement: Figure 2—figure supplement 3—source data 1. — Raw and annotated WB images. The representative western blot images for Figure 2—figure supplement 3A–D are indicated within a blue square. [file elife-89453-fig2-figsupp3-data1.zip › WB Figure 2 - figure supplement 3/Figure 2 - figure supplement 3A/Fig2-S3A Cell lysate tubulin.tif]

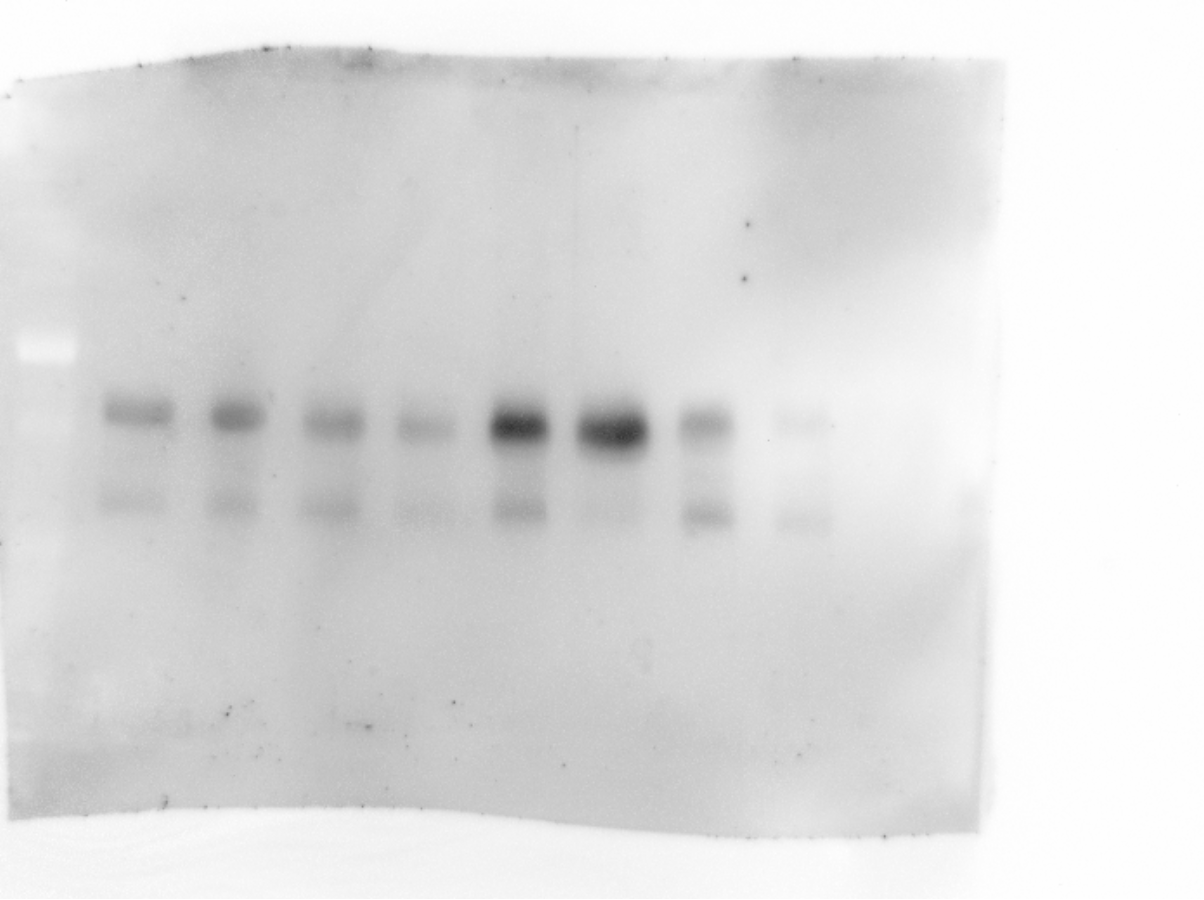

Supplement: Figure 2—figure supplement 3—source data 1. — Raw and annotated WB images. The representative western blot images for Figure 2—figure supplement 3A–D are indicated within a blue square. [file elife-89453-fig2-figsupp3-data1.zip › WB Figure 2 - figure supplement 3/Figure 2 - figure supplement 3A/Fig2-S3A Extracell Dkk3.tif]

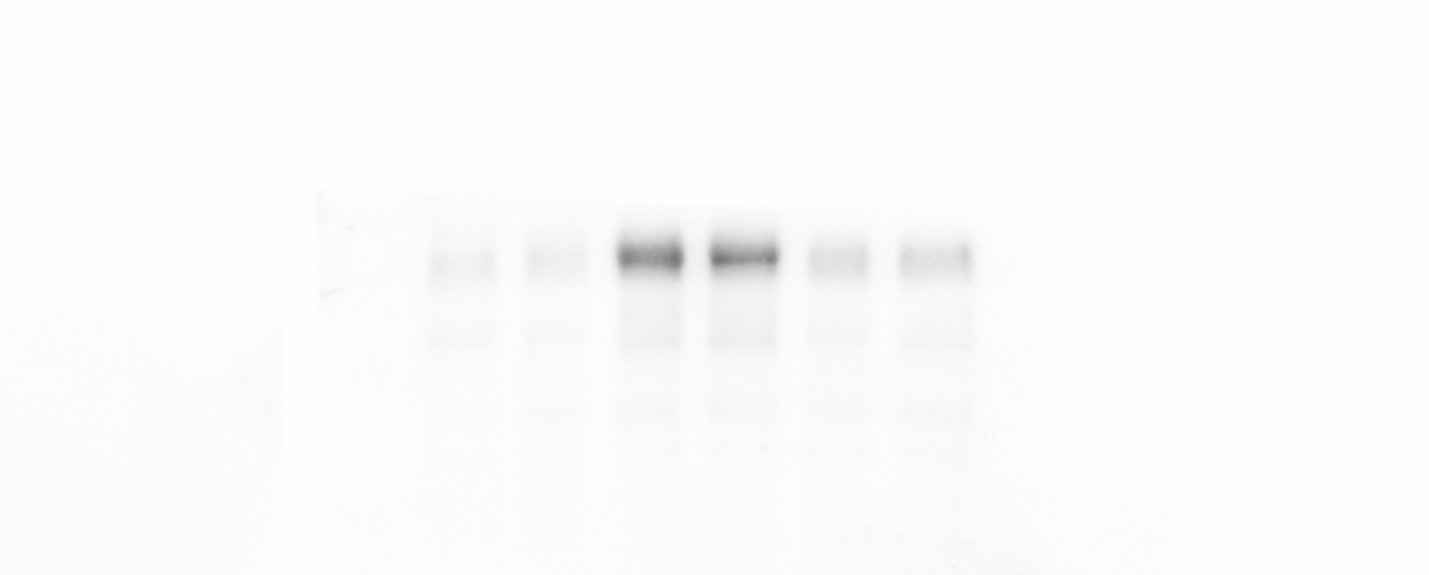

Supplement: Figure 2—figure supplement 3—source data 1. — Raw and annotated WB images. The representative western blot images for Figure 2—figure supplement 3A–D are indicated within a blue square. [file elife-89453-fig2-figsupp3-data1.zip › WB Figure 2 - figure supplement 3/Figure 2 - figure supplement 3B/Fig2-S3B Cell lysate Dkk3.tif]

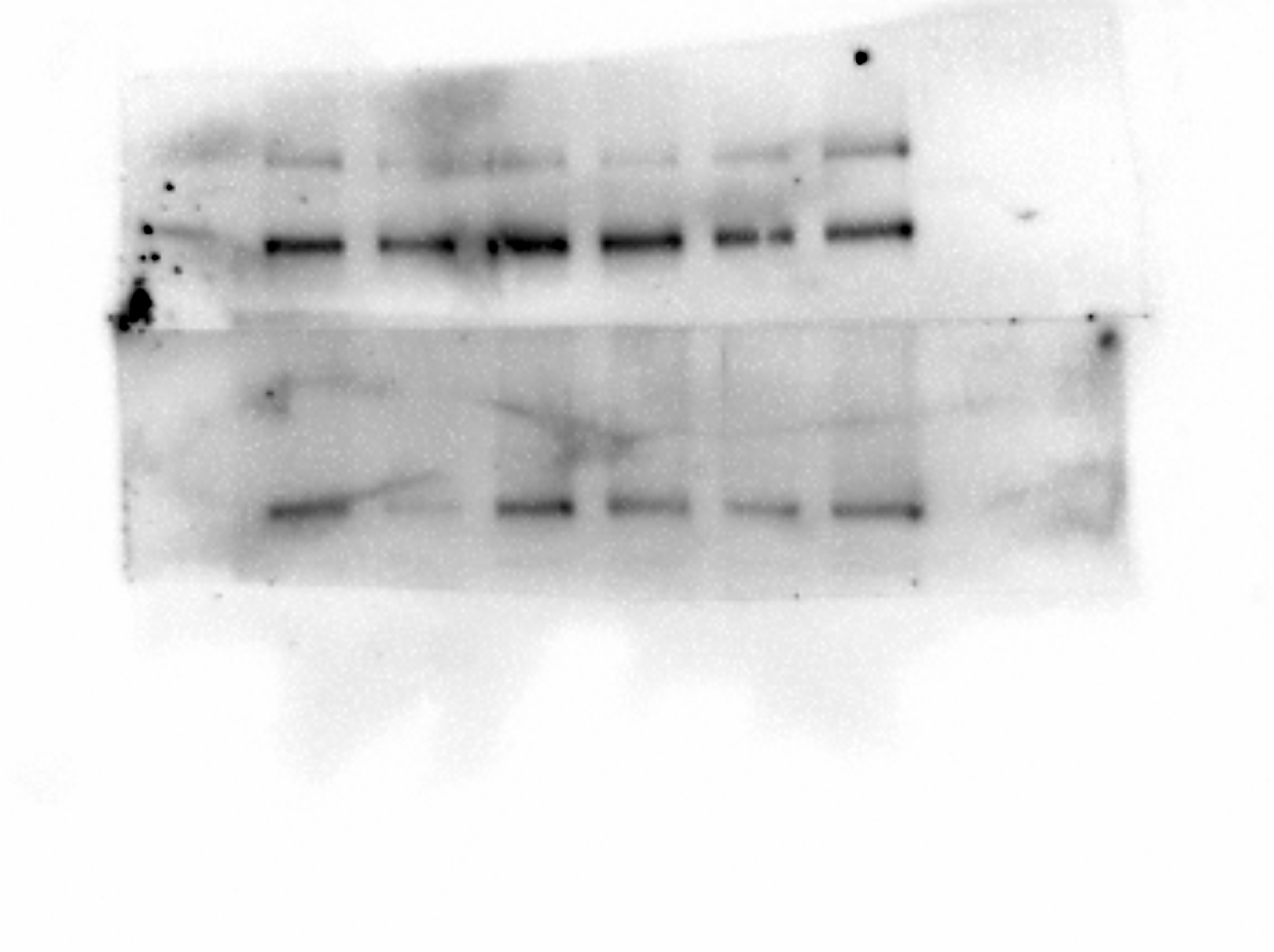

Supplement: Figure 2—figure supplement 3—source data 1. — Raw and annotated WB images. The representative western blot images for Figure 2—figure supplement 3A–D are indicated within a blue square. [file elife-89453-fig2-figsupp3-data1.zip › WB Figure 2 - figure supplement 3/Figure 2 - figure supplement 3B/Fig2-S3B Cell lysate tGluA1 pGluA.tif]

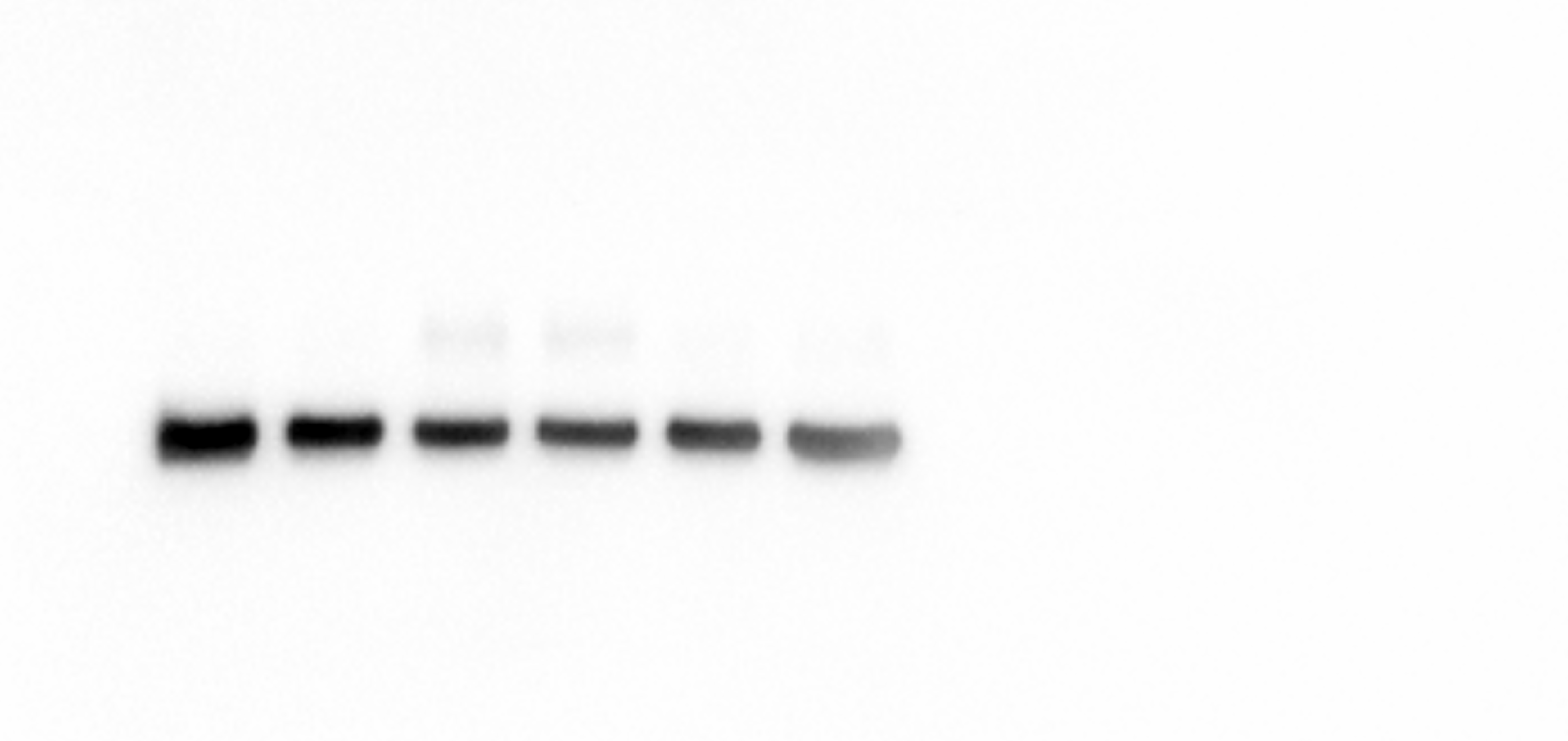

Supplement: Figure 2—figure supplement 3—source data 1. — Raw and annotated WB images. The representative western blot images for Figure 2—figure supplement 3A–D are indicated within a blue square. [file elife-89453-fig2-figsupp3-data1.zip › WB Figure 2 - figure supplement 3/Figure 2 - figure supplement 3B/Fig2-S3B Cell lysate tubulin.tif]

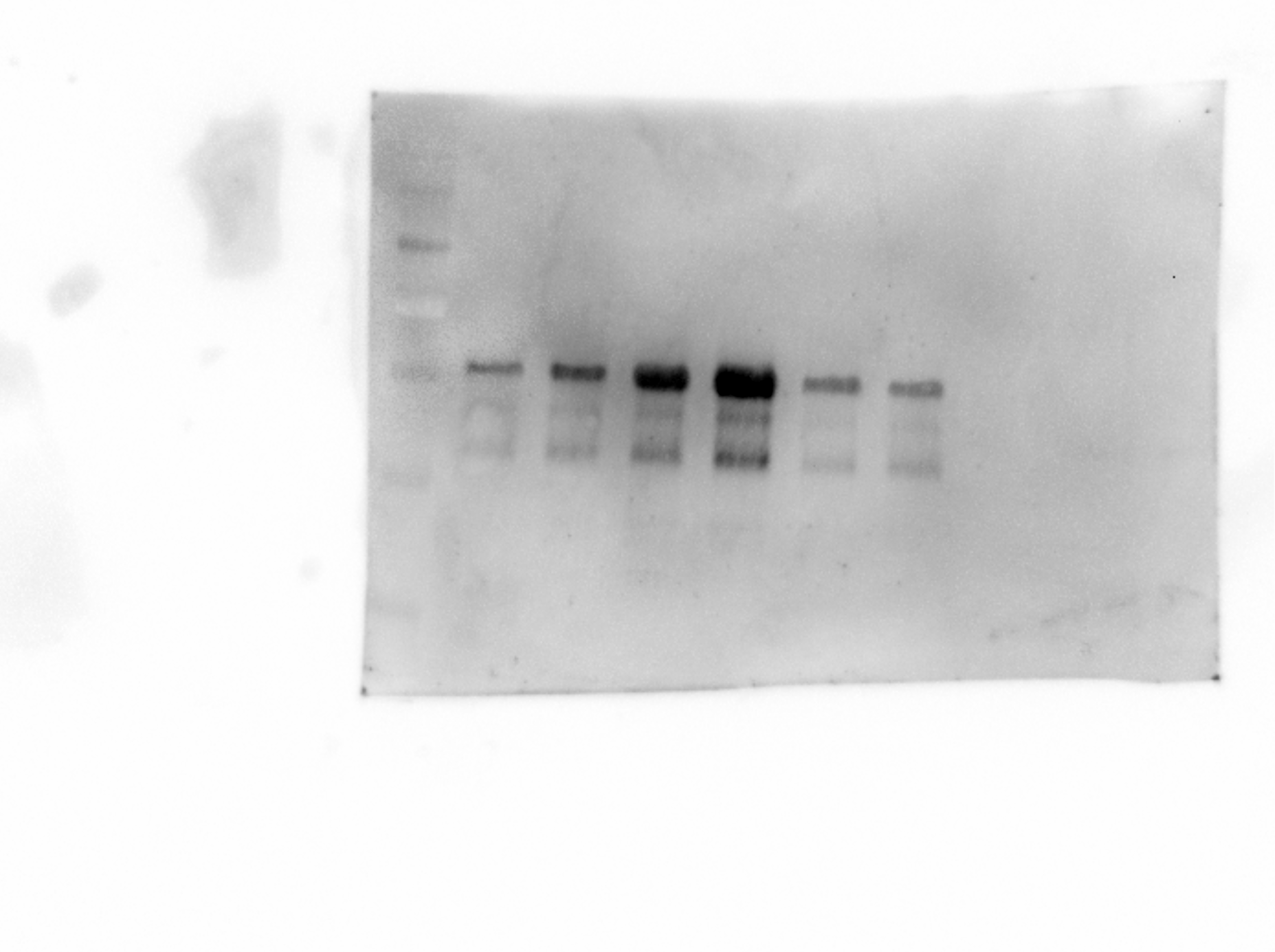

Supplement: Figure 2—figure supplement 3—source data 1. — Raw and annotated WB images. The representative western blot images for Figure 2—figure supplement 3A–D are indicated within a blue square. [file elife-89453-fig2-figsupp3-data1.zip › WB Figure 2 - figure supplement 3/Figure 2 - figure supplement 3B/Fig2-S3B Extracell Dkk3.tif]

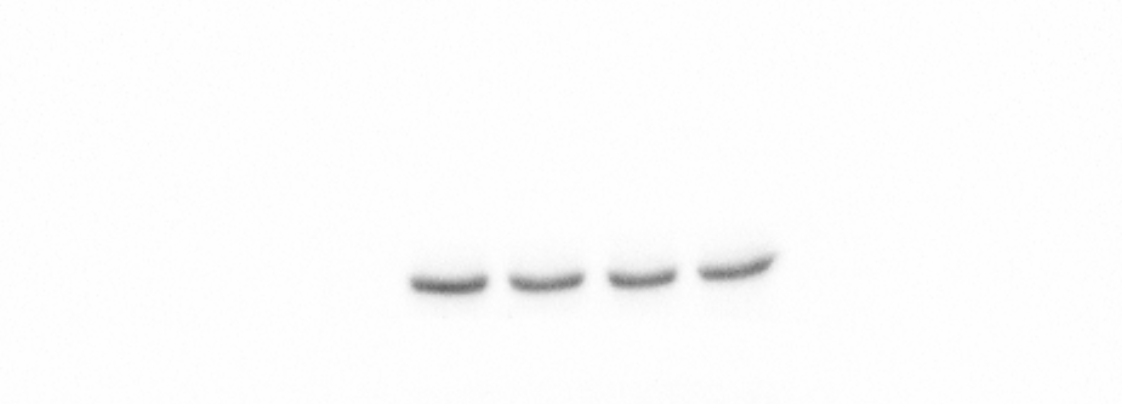

Supplement: Figure 2—figure supplement 3—source data 1. — Raw and annotated WB images. The representative western blot images for Figure 2—figure supplement 3A–D are indicated within a blue square. [file elife-89453-fig2-figsupp3-data1.zip › WB Figure 2 - figure supplement 3/Figure 2 - figure supplement 3C/Fig2-S3C Cell lysate actin.tif]

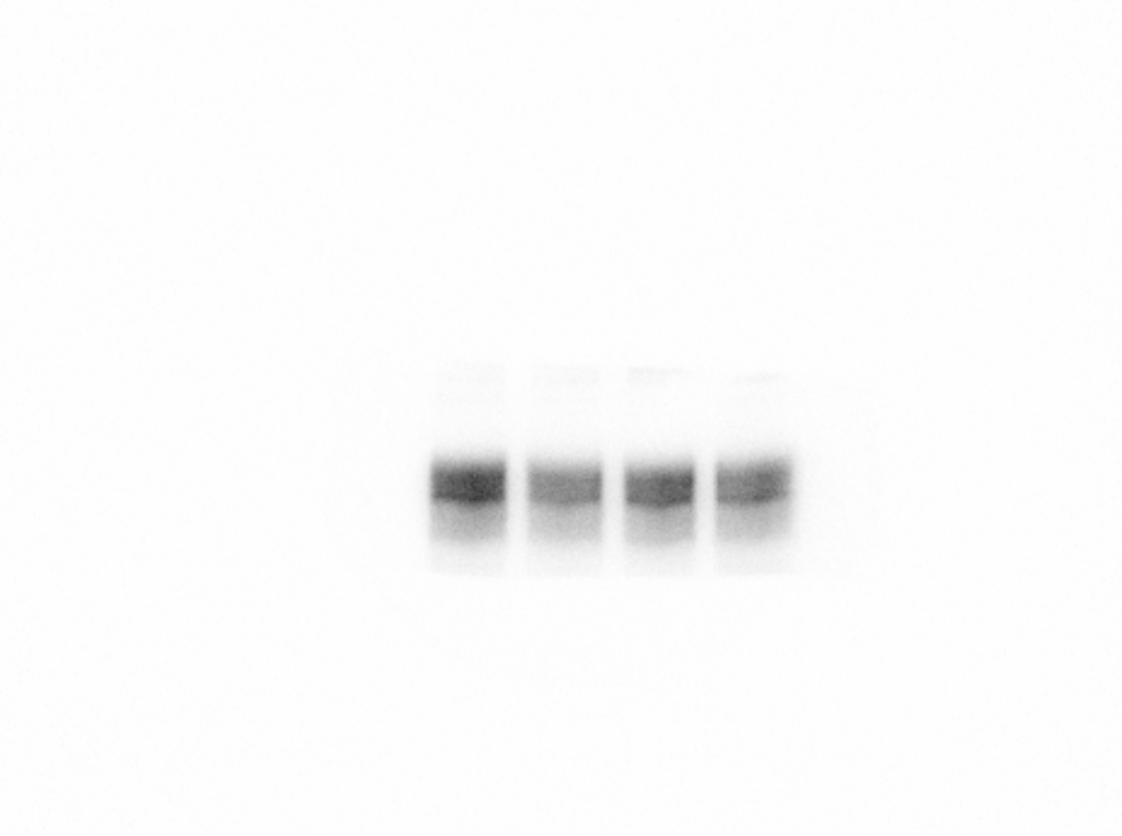

Supplement: Figure 2—figure supplement 3—source data 1. — Raw and annotated WB images. The representative western blot images for Figure 2—figure supplement 3A–D are indicated within a blue square. [file elife-89453-fig2-figsupp3-data1.zip › WB Figure 2 - figure supplement 3/Figure 2 - figure supplement 3C/Fig2-S3C Cell lysate Dkk3.tif]

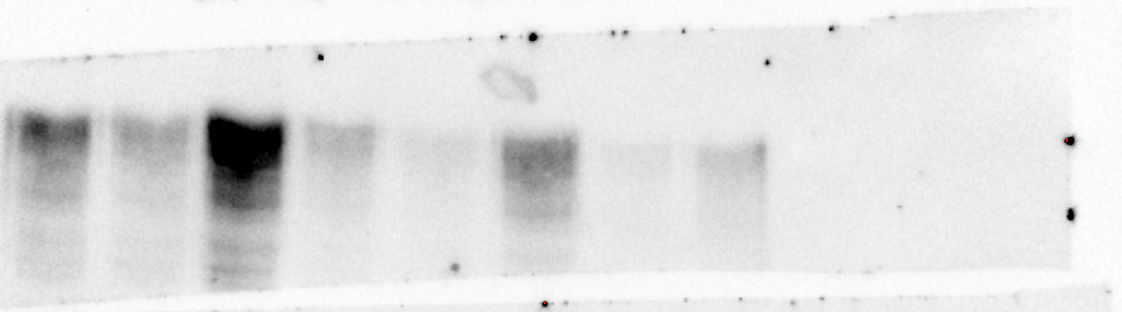

Supplement: Figure 2—figure supplement 3—source data 1. — Raw and annotated WB images. The representative western blot images for Figure 2—figure supplement 3A–D are indicated within a blue square. [file elife-89453-fig2-figsupp3-data1.zip › WB Figure 2 - figure supplement 3/Figure 2 - figure supplement 3C/Fig2-S3C Extracell Dkk3.tif]

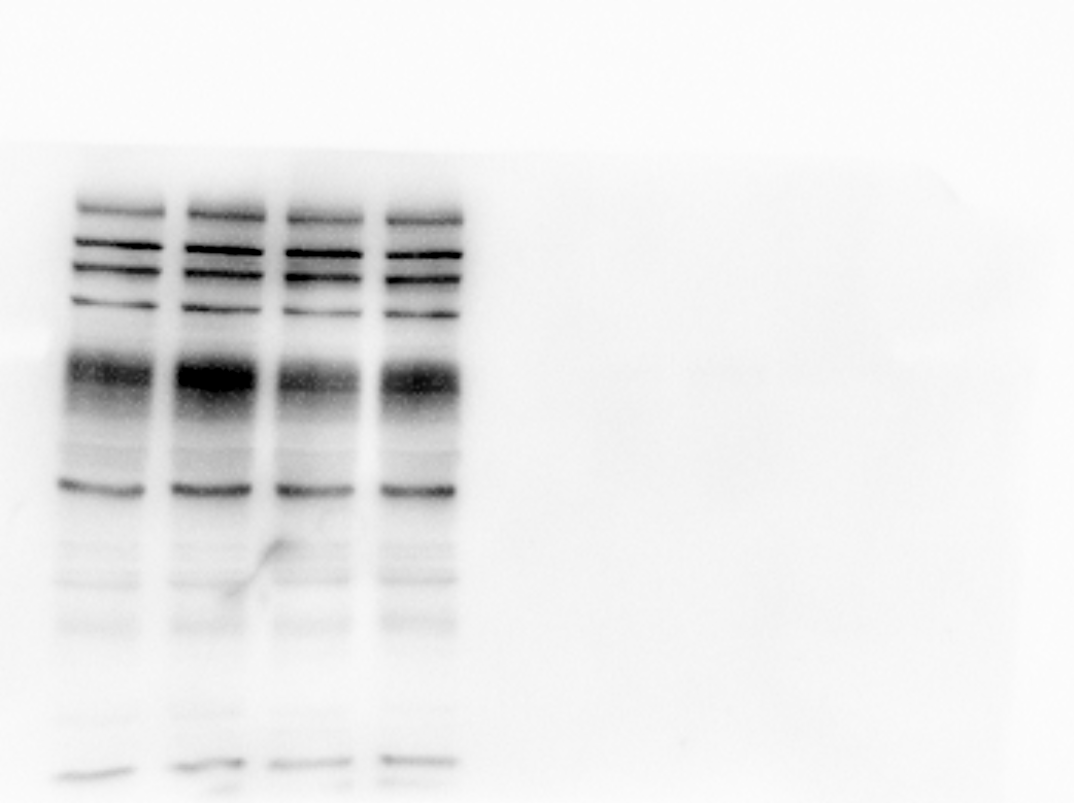

Supplement: Figure 2—figure supplement 3—source data 1. — Raw and annotated WB images. The representative western blot images for Figure 2—figure supplement 3A–D are indicated within a blue square. [file elife-89453-fig2-figsupp3-data1.zip › WB Figure 2 - figure supplement 3/Figure 2 - figure supplement 3D/Fig2-S3D Dkk3 05sec.tif]

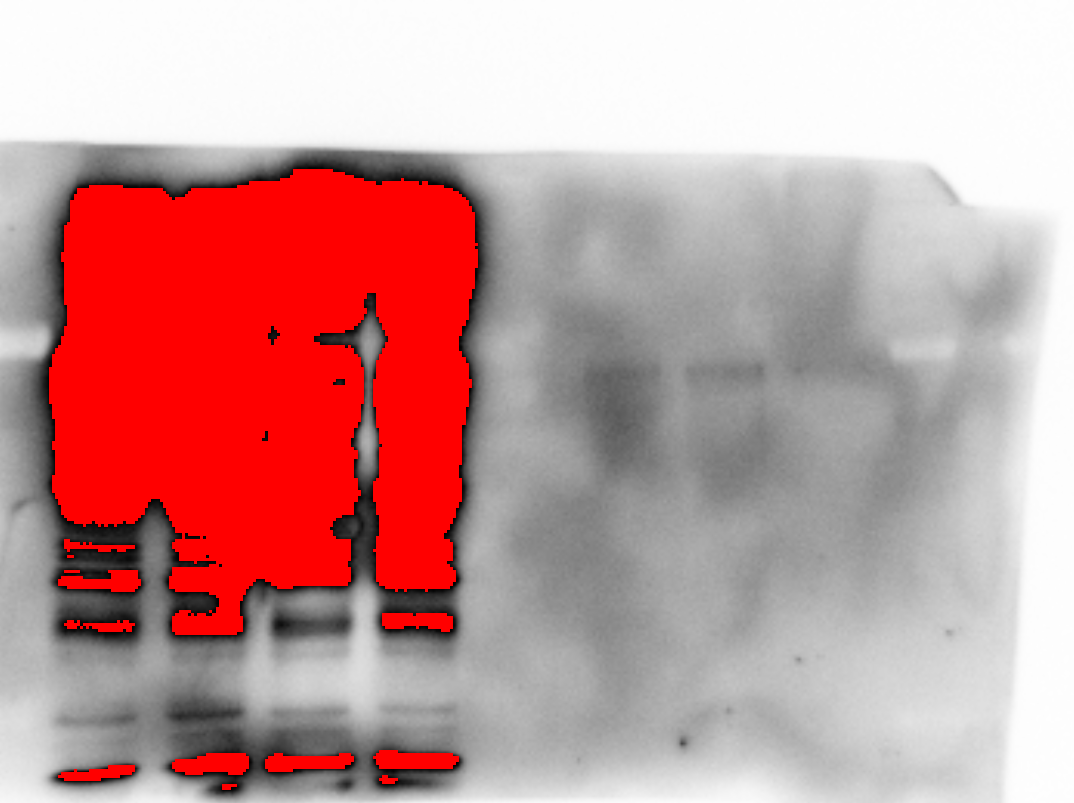

Supplement: Figure 2—figure supplement 3—source data 1. — Raw and annotated WB images. The representative western blot images for Figure 2—figure supplement 3A–D are indicated within a blue square. [file elife-89453-fig2-figsupp3-data1.zip › WB Figure 2 - figure supplement 3/Figure 2 - figure supplement 3D/Fig2-S3D Dkk3 5sec.tif]

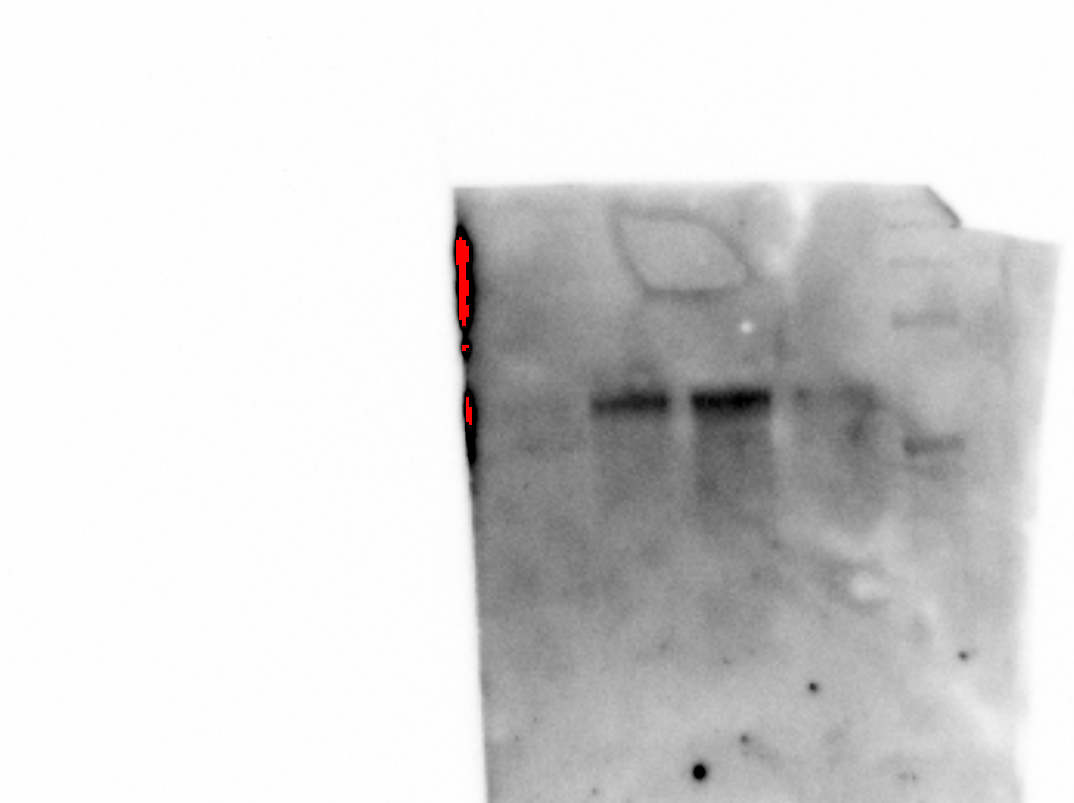

Supplement: Figure 2—figure supplement 3—source data 1. — Raw and annotated WB images. The representative western blot images for Figure 2—figure supplement 3A–D are indicated within a blue square. [file elife-89453-fig2-figsupp3-data1.zip › WB Figure 2 - figure supplement 3/Figure 2 - figure supplement 3D/Fig2-S3D Dkk3 60sec.tif]

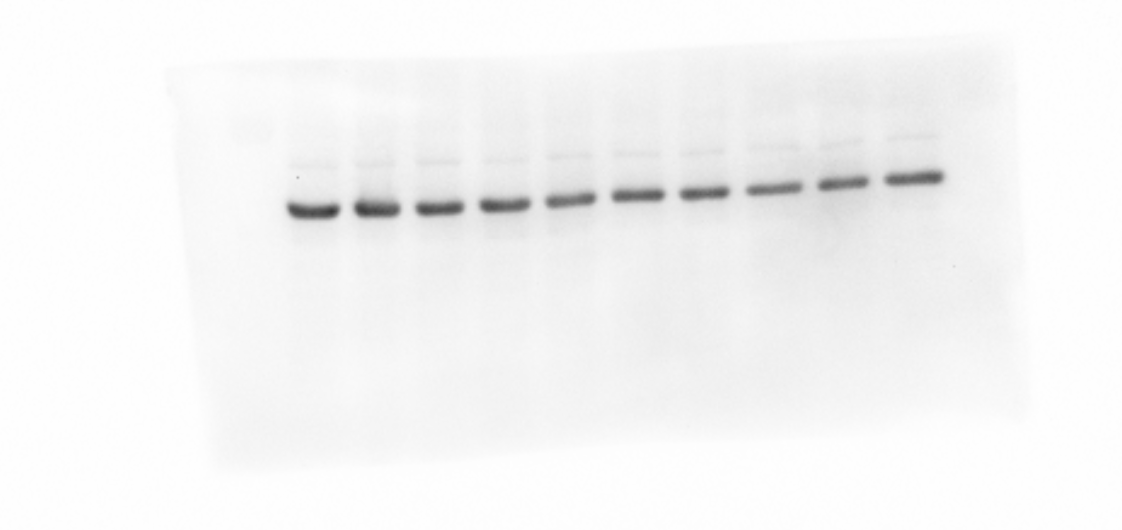

Supplement: Figure 4—source data 1. — Raw and annotated WB images. The representative western blot images for Figure 4E are indicated within a blue square. [file elife-89453-fig4-data1.zip › WB Figure 4/Fig 4E/Fig 4E actin.tif]

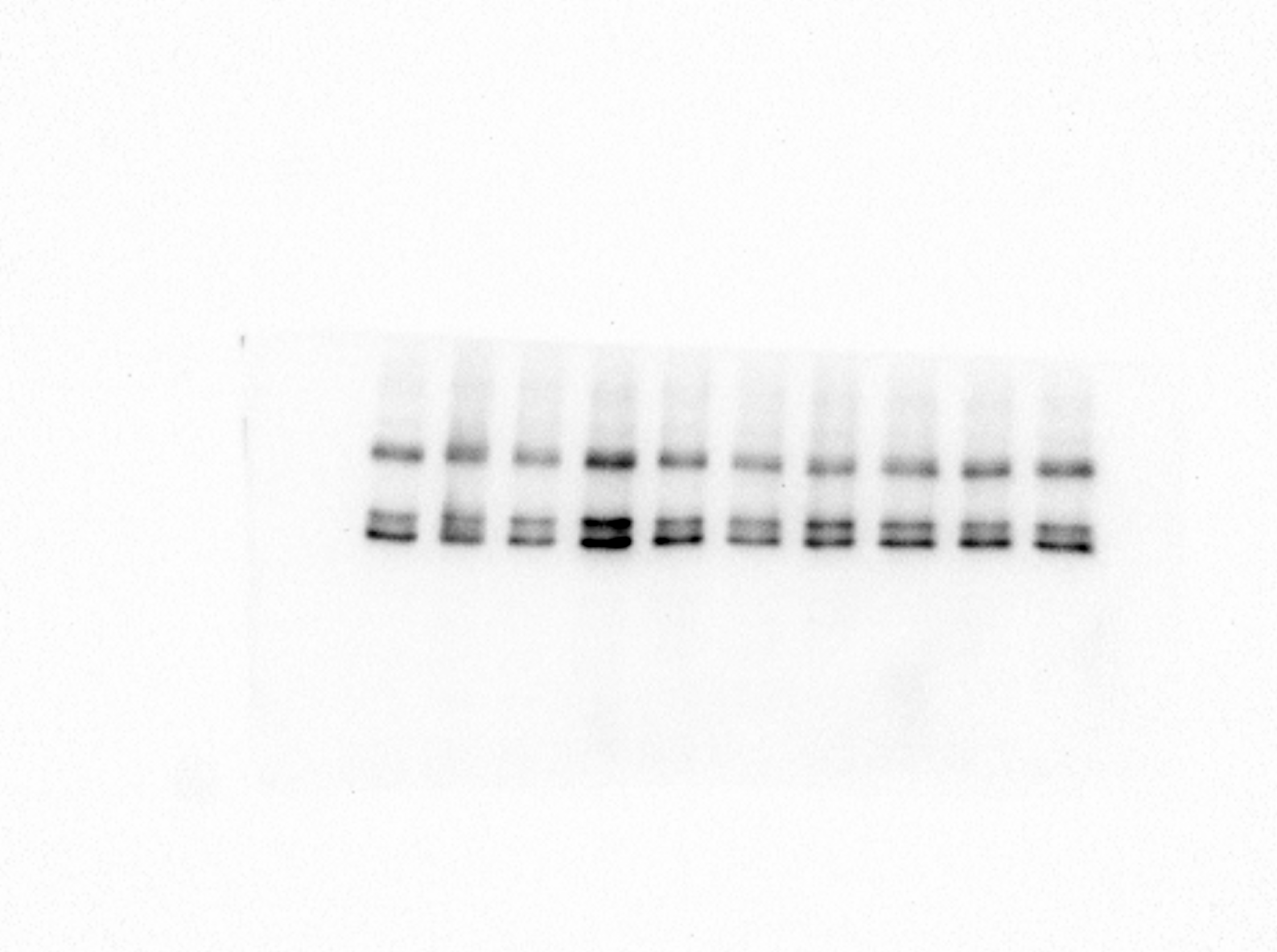

Supplement: Figure 4—source data 1. — Raw and annotated WB images. The representative western blot images for Figure 4E are indicated within a blue square. [file elife-89453-fig4-data1.zip › WB Figure 4/Fig 4E/Fig 4E pJnk.tif]

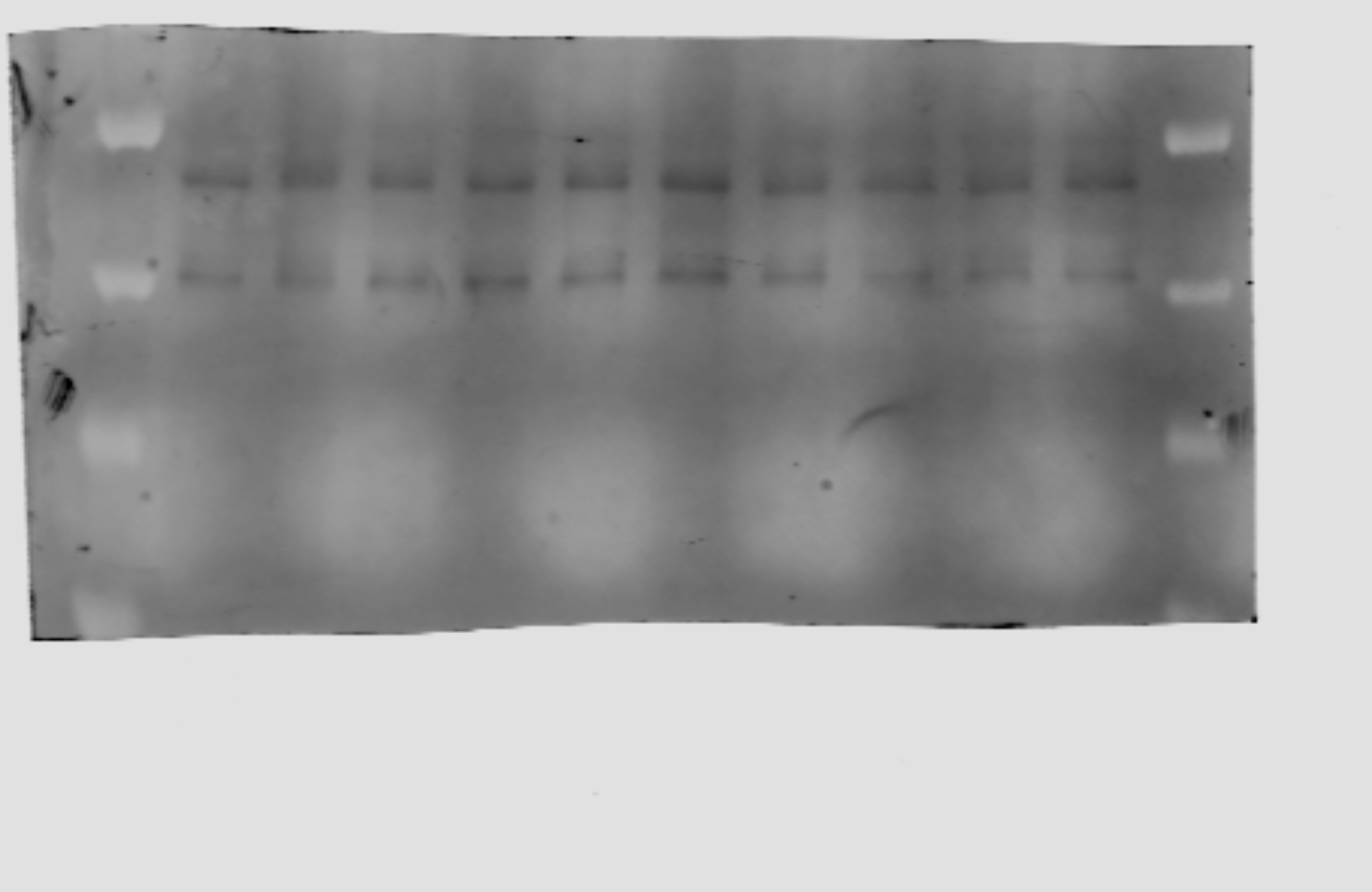

Supplement: Figure 4—source data 1. — Raw and annotated WB images. The representative western blot images for Figure 4E are indicated within a blue square. [file elife-89453-fig4-data1.zip › WB Figure 4/Fig 4E/Fig 4E tJnk.tif]

**Figure 4E**

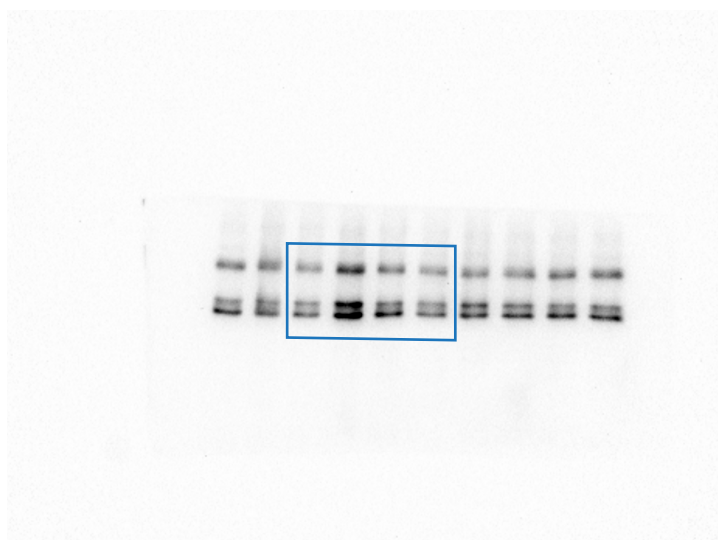

**pJnk**

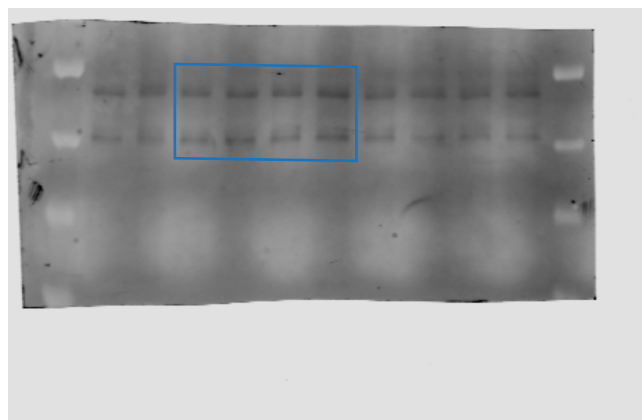

**Total Jnk**

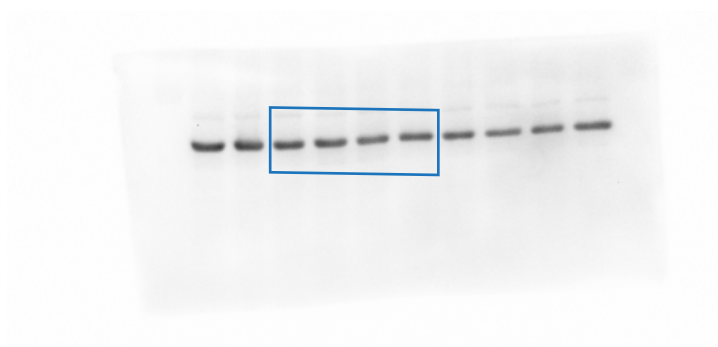

**Actin**

Supplement: Figure 4—source data 1. — Raw and annotated WB images. The representative western blot images for Figure 4E are indicated within a blue square. [file elife-89453-fig4-data1.zip › WB Figure 4/Source data_Figure 4.pdf]
