# Supplementary material for: Downregulation of Dickkopf-3, a Wnt antagonist elevated in Alzheimer’s disease, restores synapse integrity and memory in a disease mouse model
Source: eLife. 2024 Jan 29;12:RP89453. doi: 10.7554/eLife.89453 (PMC10945611; doi:10.7554/eLife.89453)
Supplement: Figure 2—figure supplement 3—source data 1. — Raw and annotated WB images. The representative western blot images for Figure 2—figure supplement 3A–D are indicated within a blue square. [file elife-89453-fig2-figsupp3-data1.zip › WB Figure 2 - figure supplement 3/Figure 2 - figure supplement 3D/Source data_Figure 2 - figure supplement 3.pdf]

Figure 2 - figure supplement 3A

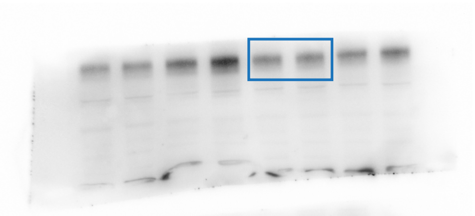

Cell lysate DKK3

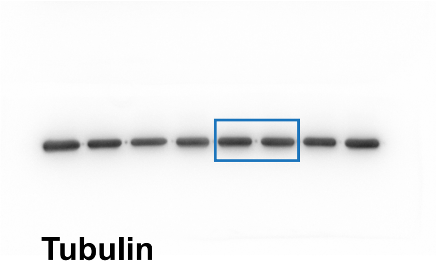

Tubulin

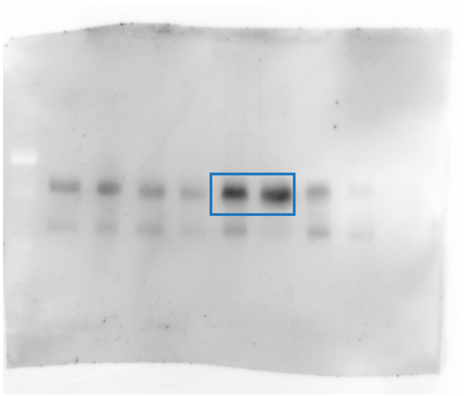

Extracell DKK3

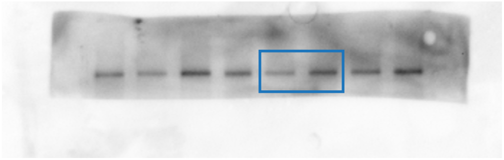

pGluA1

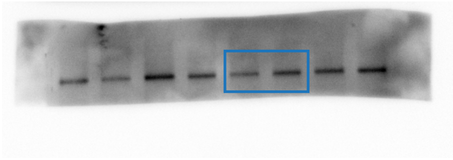

Total GluA1

Figure 2 - figure supplement 3B

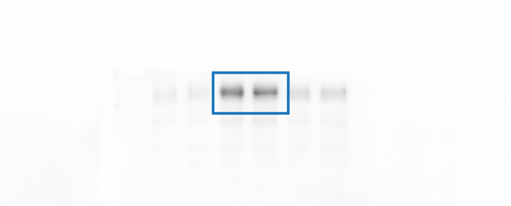

Cell lysate DKK3

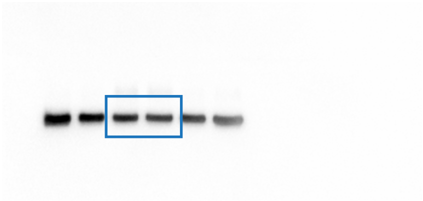

Tubulin

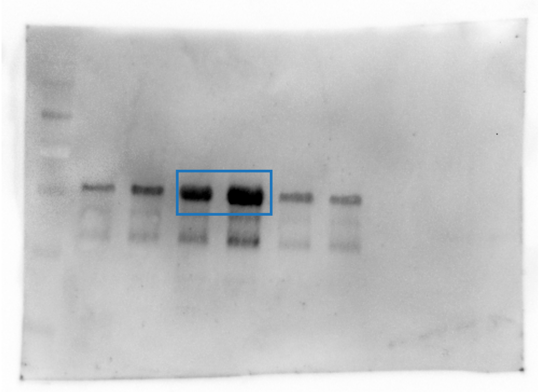

Extracell DKK3

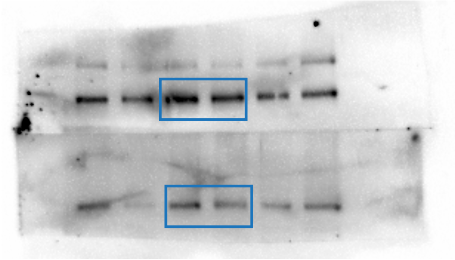

Total GluA1

pGluA1

Figure 2 - figure supplement 3C

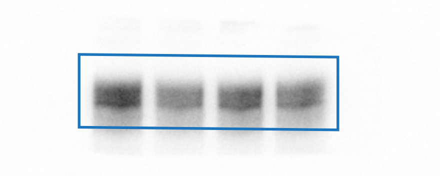

Cell lysate DKK3

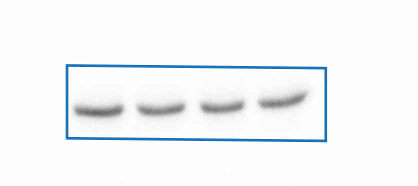

Actin

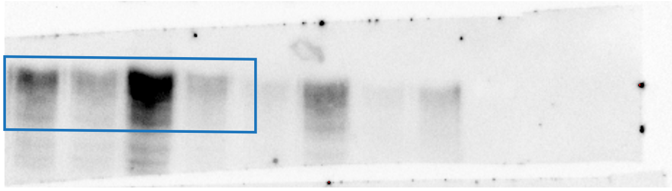

Extracell DKK3

Figure 2 - figure supplement 3D

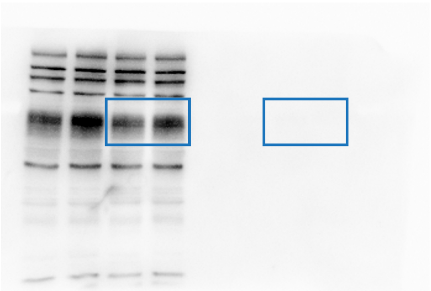

DKK3 0.5 sec

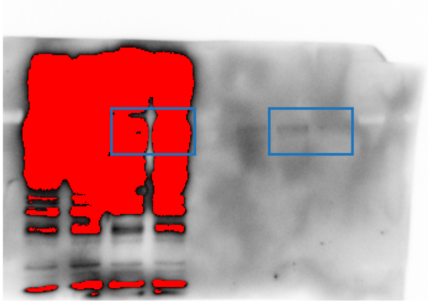

DKK3 1 sec

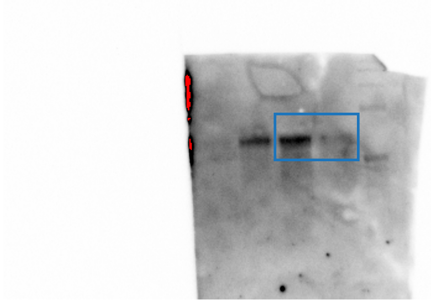

DKK3 60 sec
