# Supplementary material for: Downregulation of Dickkopf-3, a Wnt antagonist elevated in Alzheimer’s disease, restores synapse integrity and memory in a disease mouse model
Source: eLife. 2024 Jan 29;12:RP89453. doi: 10.7554/eLife.89453 (PMC10945611; doi:10.7554/eLife.89453)
Supplement: Supplementary file 1. — M=Male, F=Female, PMI = Post-mortem interval. [file elife-89453-supp1.docx]

| **Patient** | **Braak stages** | **Sex (F,M)** | **Age (years)** |
| --- | --- | --- | --- |
| 1 | 0 | F | 85 |
| 2 | 0 | F | 54 |
| 3 | 0 | F | 72 |
| 4 | 0 | M | 81 |
| 5 | 0 | F | 27 |
| 6 | 0 | F | 60 |
| 7 | 0 | M | 64 |
| 8 | 0 | F | 59 |
| 9 | 0 | M | 35 |
| 10 | 0 | M | 37 |
| 11 | 0 | M | 68 |
| 12 | 0 | M | 73 |
| 13 | 0 | F | 49 |
| 14 | 0 | M | 83 |
| 15 | 0 | M | 79 |
| 16 | 0 | F | 69 |
| **group medians (IQR)** |  | **50 : 50** | **66 (21.75)** |
| 17 | I | F | 70 |
| 18 | II | M | 87 |
| 19 | II | M | 52 |
| 20 | III | M | 79 |
| 21 | III | M | 90 |
| 22 | II | M | 70 |
| 23 | II | F | 66 |
| 24 | III | F | 77 |
| 25 | II | F | 86 |
| 26 | III | F | 91 |
| 27 | II | F | 61 |
| 28 | II | M | 83 |
| 29 | III | F | 85 |
| 30 | I | M | 66 |
| 31 | II | M | 80 |
| 32 | III | M | 88 |
| **group medians (IQR)** |  | **43.75 : 56.25** | **79.5 (17.25)** |
| 33 | VI | F | 69 |
| 34 | VI | M | 78 |
| 35 | V | F | 96 |
| 36 | VI | F | 75 |
| 37 | VI | F | 64 |
| 38 | V | M | 82 |
| 39 | V | M | 89 |
| 40 | VI | F | 79 |
| 41 | V | M | 71 |
| 42 | VI | M | 66 |
| 43 | VI | F | 78 |
| 44 | V | M | 88 |
| 45 | VI | F | 74 |
| 46 | IV | M | 86 |
| 47 | VI | F | 81 |
| 48 | V | M | 88 |

**Supplementary File 1. Human brain samples information.**
